# Enzymatic synthesis of phosphatidyl-EPA/DHA using *Candida antarctica* lipase B immobilized on mesoporous MIL-88 A

**DOI:** 10.1186/s40643-025-00959-5

**Published:** 2025-11-03

**Authors:** Yuhan Li, Guowei Wu, Zeqing Liu, Lingmei Dai, Dehua Liu, Wei Du

**Affiliations:** Key Laboratory for Industrial Biocatalysis, Ministry of Education, Department of Chemical Engineering, Tsinghua University, Beijing, 100084 China

**Keywords:** Immobilized lipase, Kinetic study, Mesoporous MIL-88A (Meso-MIL-88A), Molecular docking simulation, Phosphatidyl EPA/DHA

## Abstract

Mesoporous MIL-88A (Meso-MIL-88A) shows significant potential as an effective carrier for immobilizing large molecules such as lipases. This study investigates Meso-MIL-88A immobilized lipase for the catalysis of eicosapentaenoic acid (EPA) and docosahexaenoic acid (DHA) incorporation into soybean phosphatidylcholine. Phosphatidyl-EPA and phosphatidyl-DHA are known for their superior health benefits and have garnered significant global attention. Firstly, we developed a novel and green water-washing strategy to synthesize Meso-MIL-88A, demonstrating its significant potential as an effective carrier for immobilizing large molecules such as lipases. Nitrogen adsorption/desorption and XPS analyses revealed that water as the eluent yielded larger average pore diameters and lower sulfur residue content compared to ethanol. This optimized Meso-MIL-88A carrier was used to immobilize *Candida antarctica* lipase B (CalB@Meso-MIL-88A), which was applied to the production of phosphatidyl-EPA and phosphatidyl-DHA. The immobilized CalB@Meso-MIL-88A exhibited exceptional catalytic efficiency, achieving an unprecedented *sn*-1 positional incorporation rate of 86.8% (44.2% EPA, 42.7% DHA) with 90% EPA/DHA-ethyl ester (EE) donors, while 97% DHA-EE resulted in a record-high 90.1% DHA incorporation at the *sn*-1 position. Kinetic studies and molecular docking simulations indicated a higher substrate affinity for DHA-EE, attributed to enhanced transfer efficiency of EPA/DHA-EE in organic solvent system. This study presents the first demonstration of the potential of Meso-MIL-88A for industrial lipase immobilization via this optimized route, offering an eco-friendly and highly efficient catalytic application for nutraceutical synthesis.

**Graphical abstract:**

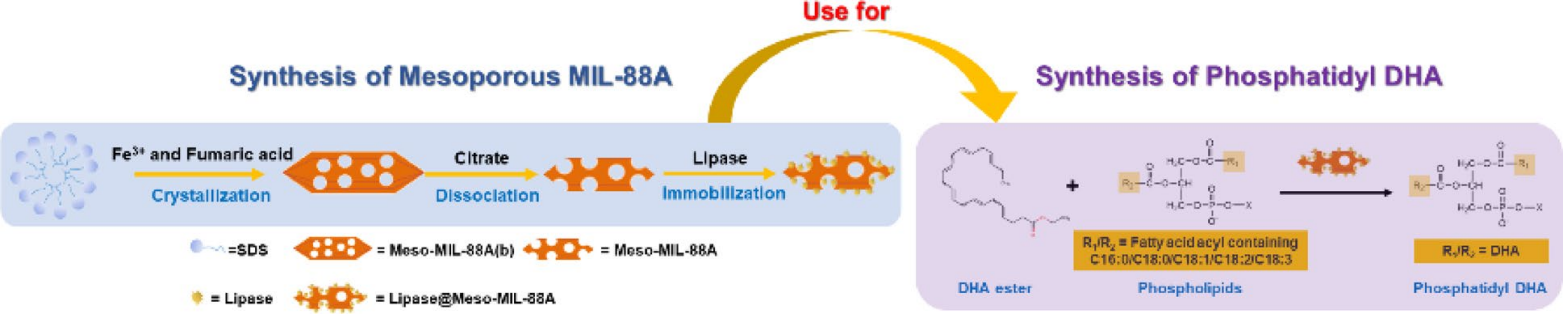

**Supplementary Information:**

The online version contains supplementary material available at 10.1186/s40643-025-00959-5.

## Introduction

Eicosapentaenoic acid (EPA) and docosahexaenoic acid (DHA) are essential omega-3 fatty acids that must be obtained through dietary intake. These acids play crucial roles in brain tissue growth (Ahmmed et al. 2023), disease prevention and treatment (Du et al. 2015; Chen et al. 2019), as well as early neural and retinal development (Hachem and Nacir 2022; Sugasini et al. 2020). Among the various forms of EPA/DHA, including ethyl esters (EE), triglyceride (TAG), and phospholipids (PLs), phosphatidyl EPA/DHA has garnered significant attention due to its superior bioavailability and antioxidative properties (Hachem and Nacir 2022; Gazquez and Larque 2021). Notably, it is the only form of EPA/DHA that can cross the blood–brain barrier (BBB) and participate in brain biochemical reactions (Ahmmed et al. 2020).

Phosphatidyl EPA/DHA can be synthesized through several methods, such as physical extraction from natural products, microbial fermentation, chemical synthesis, and enzymatic synthesis (Zhang et al. 2024). Enzymatic synthesis stands out for its high efficiency, mild reaction conditions, and enhanced safety (He et al. 2022). Common approaches include enzyme-mediated transesterification of phospholipids with EPA/DHA-rich esters and acidolysis of phosphatidylcholine (PC) with EPA/DHA (Wang et al. 2019; Marsaoui et al. 2015). However, free EPA/DHA are easy to oxidize during the reactions and derived from their EE form, causing a complex preparation process and difficulty preserving for a long time (Vikbjerg et al. 2005; Xi et al. 2016; Li et al. 2016). Therefore, transesterification of PC with EPA/DHA esters is considered a more viable method for synthesizing phosphatidyl EPA/DHA. For example, Novozym 435 was used to transesterify phosphatidylcholine (PC) with EPA/DHA-EE over a 48-h period, achieving a total incorporation rate of 45.6% EPA/DHA into PC (Chojnacka et al. 2017). Zhang et al. (Zhang et al. 2023a) reported a 39.1% total incorporation of DHA into PC using phospholipase A_1_.

Metal–organic frameworks (MOFs) have attracted attention due to their exceptional physical and chemical properties, such as controllable pore size, high specific surface area, functional surface modification, and versatile functionalities (Wang et al. 2020; Kim et al. 2001; Lian et al. 2017). However, the microporous nature of MOFs limits the accessibility of large molecules and hinders diffusion, restricting their application in lipase immobilization (Cai and Jiang 2017; Hu et al. 2021; Zhou et al. 2022; Cheng et al. 2023). The development of mesoporous MOFs has emerged as a promising strategy to address these challenges and has become a focal point of research (Cai and Jiang 2017; Zhang et al. 2015; Ren et al. 2019). Additionally, the stability of MOFs towards water and acid is critical for lipase immobilization, given that the catalytic environment often involves fatty acids and water. Selecting pristine MOFs with inherent tolerance to both acid and water may eliminate the need for further modification, making them more economical. According to the hard-soft acid–base (HSAB) theory, MIL-88A, based on Fe (III) trimers interconnected through fumaric acid dicarboxylates, exhibits both stability and economy, making it a potential carrier for enzyme immobilization (Mellot-Draznieks et al. 2005; Yuan et al. 2018).

Meso-MIL-88A demonstrates significant potential as a novel immobilized carrier due to its robust tolerance to acidic aqueous systems and expanded pore structure, which facilitates the immobilization of large-sized lipases (Zhang et al. 2018). These properties position it as a promising platform for lipase-mediated synthesis of phosphatidyl-EPA/DHA. Notably, its well-established stability in water-rich environments offers a unique opportunity to reconsider the conventional synthesis protocol, which traditionally depends on organic solvents such as ethanol for washing steps. We hypothesized that this aqueous stability could be leveraged to develop a greener and more efficient washing strategy. Specifically, we proposed that water—owing to its superior solubility for SDS—could more effectively remove the templating surfactant, thereby yielding a cleaner and more accommodating microenvironment for subsequent enzyme immobilization. In this work, superior Meso-MIL-88A carriers were prepared using different elution agents (ethanol and water). The optimized carrier was subsequently employed for lipase immobilization. The resulting CalB@Meso-MIL-88A biocatalyst was then applied to produce phosphatidyl-EPA/DHA. Different reaction media was comparatively investigated to maximize total EPA/DHA incorporation into PC. Finally, the transesterification kinetics of CalB@Meso-MIL-88A and molecular docking simulations between CalB and EPA/DHA-EE substrates were conducted to elucidate the reaction mechanisms governing phosphatidyl-EPA/DHA synthesis.

## Material and methods

### Materials

*Candida antarctica* lipase B (CalB) and Lipozyme TL IM were obtained from Novozymes (Denmark). Fumaric acid (FA, 99%) and sodium dodecyl sulfate (SDS, 92.5–100.5%) were bought from Macklin Co., Ltd (China). FeCl_3_·6H_2_O (99%) and thin layer chromatography (TLC) plates were bought from Shanghai Titan Technology Co., Ltd (China). Sodium citrate (98%) was bought from Heowns Biochem Technologies Co., Lt (China). Tributyrin (98%) and soybean phosphatidylcholine (98%) were bought from Bidepharm Co., Lt (China). 90% EPA and DHA ethyl ester (50% EPA-EE and 40% DHA-EE) was bought from Shaanxi Kanghe Pharmaceutical Co., LTD (China). 80% and 97% DHA ethyl ester (DHA-EE) were bought from Shanghai Tauto Biotech Co., Ltd (China). Karl Fischer’s reagent and molecular sieve 4A were bought from Shanghai Aladdin Biochemical Technology Co., LT (China). Ethanol (95%) was bought from Beijing Tongguang Fine Chemical Co., LT (China). Anhydrous ethanol, chloroform, anhydrous methanol, potassium phosphate dibasic, potassium phosphate monobasic, and other chemicals were analytically pure in this work.

### Synthesis of meso-MIL-88A

**Fig. 1:**
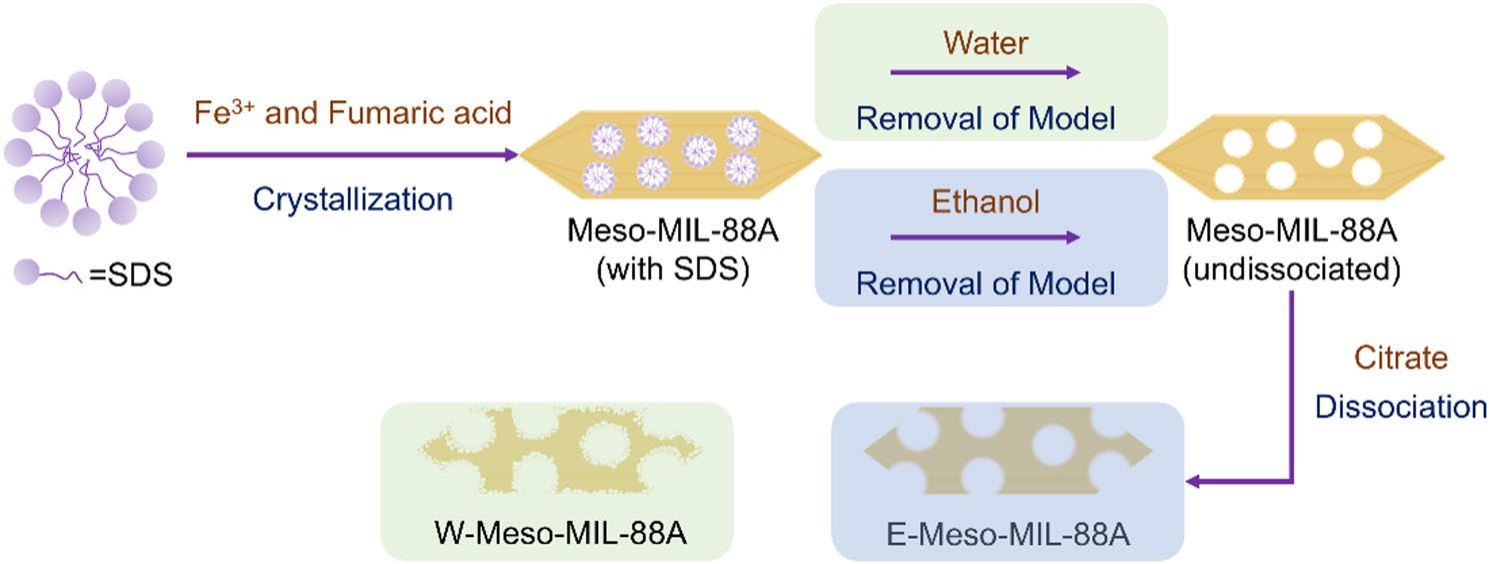
Schematic of the synthesis of E-Meso-MIL-88A and W-Meso-MIL-88A

There was an improvement in the preparation method of mesoporous MIL-88A (Meso-MIL-88A) compared with our previous work (Li et al. 2024) as described in Support Information and Fig. 1. The preparation of mesoporous MIL-88A was achieved using SDS as a soft templating agent. Deionized water rather than ethanol was used to wash the MOFs precipitates to remove SDS and any unreacted precursor solution efficiently. Mesoporous MIL-88A was washed with either ethanol or water, yielding the carriers designated E-Meso-MIL-88A and W-Meso-MIL-88A, respectively.

### Synthesis and specific activity assay of CalB@Meso-MIL-88A

50 mg of Meso-MIL-88A was added to 800 μL deionized water in a plastic centrifuge tube. The carrier was uniformly dispersed by ultrasonication for 15 min. Subsequently, 200 μL of *Candida antarctica* lipase B (CalB) solution (21.30 mg/mL) was added to the mixture, and the mixture was then incubated in a thermostatic shaker at 40 °C and 200 rpm for 4 h. This concentration was selected based on optimization experiments that provided the best balance of high activity recovery and immobilization efficiency (Fig. S1). The immobilized lipase was then collected by centrifugation at 6000 rpm for 5 min and washed once with water. Then, the immobilized lipases were lyophilized overnight and stored at 4 °C for further analysis, while the supernatants were stored at − 20 °C for further testing.

The determination of enzyme loading, activity, and activity recovery are described in the Supporting Information.

### CalB@Meso-MIL-88A-catalyzed synthesis of phosphatidyl EPA/DHA under different conditions

Under solvent-free conditions, the enzymatic transesterification of soybean phosphatidylcholine (PC) with EPA/DHA ethyl ester (EPA/DHA-EE) or DHA ethyl ester (DHA-EE) was conducted in a 2 mL plastic centrifuge tube placed in a thermostatic shaker at a specific temperature (45, 50, 55 °C) and 250 rpm. The reaction mixture comprised 0.075 mmol of soybean PC, 90% EPA/DHA-EE (50% EPA-EE and 40% DHA-EE) or 80% DHA-EE or 97% DHA-EE with different molar ratios (the molar ratio of ethyl ester to PC was *x*, *x*:1 = 4:1/8:1/16:1/32:1), and 450/900/1800 U of immobilized lipase enzymatic activity. The analysis of fatty acid composition by gas chromatograph (GC) is described in Support Information.

Under different solvent systems, the CalB@Meso-MIL-88A-catalyzed transesterification was conducted in a 15 mL pressure tube with an internal screw plug to prevent organic solvent volatilization. The system contained 0.26 mmol PC, 2.34 mmol (molar ratio was 9:1) EPA/DHA EE or DHA-EE, 1.5 mL organic solvent (pure hexane, or hexane: tert-butanol = 1:1, or hexane: butanone = 1:1) and 900 U of immobilized lipase enzymatic activity. The molecular sieves (MS) used in some systems were 1 g 4A molecular sieves activated by vacuum at 150 °C for 2 h. At different reaction times, a 150 μL sample was withdrawn. Among them, a 100 μL sample was used to determine the water content of the system measured by a moisture meter. Then, the remaining 50 μL sample was used to assess the total incorporation of EPA/DHA into PC. The solvent was removed from the sample by spin evaporation under 60 °C for 15 min. The analysis of fatty acids in the *sn*-1 and *sn*-2 position of PC is described in Support Information. “Total incorporation” refers to the combined content of EPA and DHA across both *sn*-1 and *sn*-2 positions of PC. The “positional incorporation” or “distribution” specifies the percentage of a particular fatty acid (e.g., DHA) residing at the *sn*-1 or *sn*-2 position.

### Kinetic studies of CalB@Meso-MIL-88A

Kinetic studies for the transesterification of soybean PC with EPA-EE and DHA-EE by CalB@Meso-MIL-88A were determined using the Michaelis–Menten equation. Under solvent-free system, the concentration ([S]) of EPA-EE varied from 1132 to 1335 mM, and [S] of DHA-EE varied from 840 to 920 mM, respectively. 0.075 mmol soybean PC and 360 U CalB@Meso-MIL-88A were added into the 2 mL reaction system. For solvent system, the concentration ([S]) of EPA-EE varied from 368 to 545 mM, and [S] of DHA-EE varied from 273 to 404 mM, respectively. 0.26 mmol soybean PC, 1.5 mL solvent, and 360 U CalB@Meso-MIL-88A were added into reaction system. The mixture was incubated under 55 °C and 250 rpm for 30 min.

### Molecular docking simulation of CalB with EPA-EE and DHA-EE

Molecular docking simulations were performed with CHARMm force field, using Discovery Studio 2019 (Accelrys Inc.). The CalB crystal structure was from the Protein Data Bank (PDB entry: 1TCA; resolution of 1.55 A). Flexible docking was chosen where both the receptor and ligands were flexible to improve docking accuracy. The strategy involved the generation of initial ligand orientations (EPA-EE or DHA-EE) in the protein’s active site, followed by molecular dynamic and final minimization. To compare the docking between CalB and EPA-EE with that of DHA-EE, the situation with the highest LidDockScore was considered.

### Characterization

Field-emission scanning electron microscope (SEM) images were acquired using a Hitachi Limited SU-8010 at an acceleration voltage of 10.0 kV. Transmission electron microscope (TEM) images were captured using a Hitachi Limited H-7650B at an operating voltage of 120 kV. The samples’ powder X-ray diffraction (PXRD) analysis was conducted using the Rigaku MiniFlex X-ray diffractometer with Cu Kα radiation at a 10 °C/min scan rate. Attenuated total reflection Fourier transform infrared spectroscopy (ATR-FTIR) data were obtained using a Bruker V70 instrument. Thermo Fisher ESCALAB 250Xi conducted X-ray Photoelectron Spectroscopy (XPS). N_2_ adsorption–desorption isotherms were measured at 77 K using an SI-MP analyzer from Beijing Kangta Technology Co., Ltd., with the samples degassed at 40 °C for 11 h before the measurements. The water content of the system was measured by moisture meter Mettler Toledo V20 using the Carl Fischer method.

## Results and discussion

### Characterization and immobilization performance of W-MIL-88A and E-MIL-88A

MIL-88A exhibits high stability towards acids and water (Hmoudah et al. 2023), making it a promising candidate for practical applications, especially in high-value oil conversion. However, the microporous nature of MIL-88A limits its application in lipase immobilization (Cai and Jiang 2017; Hu et al. 2021; Zhou et al. 2022; Cheng et al. 2023). To expand the pore sizes of MIL-88A, we synthesized mesoporous MIL-88A (Meso-MIL-88A) using a soft-template method followed by a citrate dissociation strategy as shown in Fig. 1.

In our previous work (Li et al. 2024), ethanol was used to remove SDS and unreacted precursor after the 24-h reaction. However, some SDS micelles could not be thoroughly removed through ultrasonic treatment in ethanol and remained in Meso-MIL-88A, which inevitably expanded the secondary structure of lipase and caused the loss of enzyme activity (Zhang et al. 2018). Moreover, the high production costs and severe pollution caused by the large-scale washing of ethanol in industrial applications set up barriers to its practical applications. Therefore, there is a need to better the removed template strategy and validate the effects of the removed template method. High stability towards the water of MIL-88A, higher solubility of SDS in water (15 g/100 mL, 20 °C) than that in ethanol (7.5 g/100 mL, 20 °C), and lower industrial application costs all make it rational to select deionized water as a more sensible eluent alternative in the place of ethanol.

**Fig. 2:**
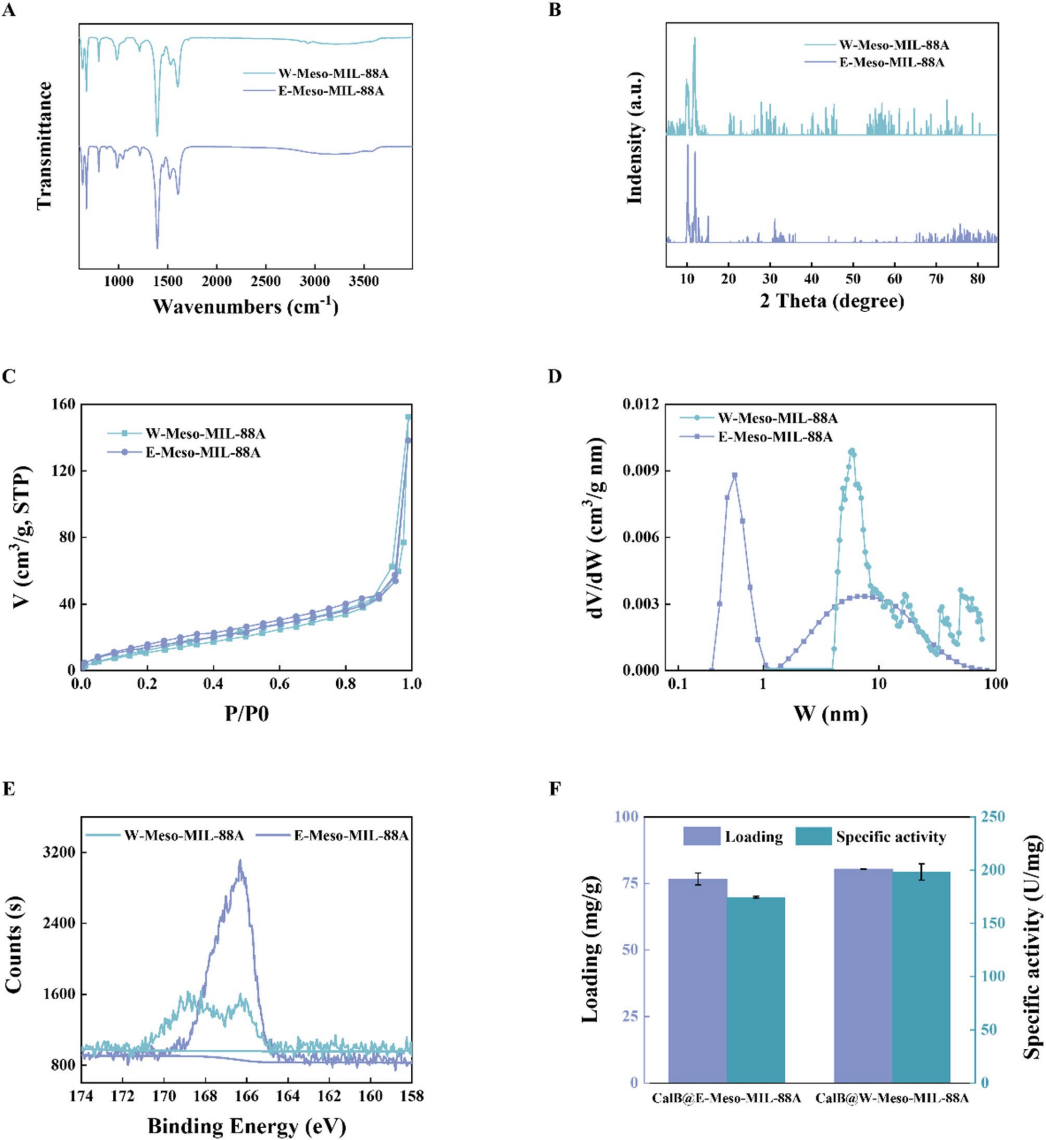
Characterization and immobilization performance of W-MIL-88A and E-MIL-88A. **A** ATR-FTIR pattern, **B** XRD pattern, and **C** N_2_ adsorption–desorption isotherms of E-Meso-MIL-88A and W-Meso-MIL-88A. **D** Corresponding pore distributions of two materials mentioned above. The samples were degassed at 40 °C for 11 h before the measurements. The computational model of pore size distribution was N_2_@77 NLDFT(SD3) model (slit holes). **E** XPS pattern (S2p) of W-Meso-MIL-88A and E-Meso-MIL-88A. **F** Loading and specific enzyme activity of immobilized CalB on W-Meso-MIL-88A and E-Meso-MIL-88A

To validate the effects of water on removing templates, we maintained experimental conditions unchanged based on the previous experiment, and the only independent variable was the type of eluent (deionized water or ethanol) utilized to remove templates. As the TEM and SEM images (Fig. S2) show, part of Meso-MIL-88A washed by water (W-Meso-MIL-88A) exhibited an irregular crystal structure, increasing the roughness of the MIL-88A surface than that of Meso-MIL-88A washed by ethanol (E-Meso-MIL-88A). This phenomenon can be attributed to the higher solubility of sodium citrate in residual water on the surface of Meso-MIL-88A than ethanol, which enhances citrate dissociation, as previously observed. The ATR-FTIR spectra depicted in Fig. 2A revealed the same structural characteristics of the two MIL-88A samples. The bands at 1392 cm^−1^ and 1603 cm^−1^ were assigned to the symmetric and asymmetric vibration modes of the fumarate carboxyl group (-COOH), indicative of the MOF bridging ligand C = O group vibration. The bands observed at 1213 cm^−1^, 982 cm^−1^, and 671 cm^−1^ corresponded to the stretching vibration of C–C, the bending vibration of C-H, and the stretching vibration of Fe–O, respectively (Ren et al. 2020; Fu et al. 2020). The XRD pattern (Fig. 2B) indicated that both W-Meso-MIL-88A and E-Meso-MIL-88A exhibited diffraction peaks near two theta = 10.8° and 12.0°, corresponding to the (100) and (101) planes of MIL-88A respectively. These characteristic peaks were consistent with those reported in the literature (Andrew Lin et al. 2015), confirming the structure of MIL-88A. Notably, additional crystal planes appeared in W-Meso-MIL-88A compared to E-Meso-MIL-88A as it exhibited more diffraction peaks whose two theta ranged from 20.0° to 70.0°, which probably corresponded to its rough surface shown in its SEM images. The consistent FTIR spectra and XRD pattern of the two MIL-88A samples along with that of the pristine microporous MIL-88A (Fig. S3) suggested that the change of eluent from ethanol to water had no damage to the chemical structure or crystal composition of MIL-88A.

To confirm the character of W-Meso-MIL-88A as support for lipase immobilization, the external surface area and surface residue of S element of W-Meso-MIL-88A were measured and compared with that of E-Meso-MIL-88A. The N_2_ adsorption–desorption isotherms, along with the corresponding pore size distributions calculated by the N_2_@77 NLDFT(SD3) model (slit holes) of E-Meso-MIL-88A and W-Meso-MIL-88A were analyzed to confirm the effects of removed template method on the external surface and the average pore diameter (Fig. 2C, D and Table 1). The external surface area of W-Meso-MIL-88A, calculated using the *t*-plot method, was determined to be 52 m^2^/g, near the 59 m^2^/g of E-Meso-MIL-88A we measured in our previous work (Li et al. 2024). However, the average pore diameter of W-Meso-MIL-88A was 18 nm, twice as large as the 9 nm of E-Meso-MIL-88A, indicating potential applicability for W-Meso-MIL-88A using in lipase immobilization. For the residue of the S element, the XPS pattern (Fig. 2E) demonstrated some differences between the two MOFs. The PP Atomic of S2p was 2.48% of W-Meso-MIL-88A, apparently lower than the 9.52% of E-Meso-MIL-88A, indicating that W-Meso-MIL-88A had less surface residue of S element. Considering that only SDS in the system contains S element, the XPS pattern revealed that deionized water was more effective in removing template SDS than ethanol, causing less adverse effect on immobilized lipase activity. The performance of W-Meso-MIL-88A and E-Meso-MIL-88A as immobilized support was then compared, as shown in Fig. 2F. Both loading and enzyme specific activity of W-Meso-MIL-88A increased to a certain extent compared with E-Meso-MIL-88A, mainly owing to the larger average pore diameter of support and less surface residue of SDS. The immobilized lipase CalB on W-Meso-MIL-88A used in the subsequent transesterification reaction of soybean PC and EPA/DHA-EE was named CalB@Meso-MIL-88A for convenience.

**Table 1:** External surface area and average pore diameter of E-Meso-MIL-88A and W-Meso-MIL-88A

MOFs	External surface area (cm^2^/g)	Average pore diameter (nm)
E-Meso-MIL-88A	59	9
W-Meso-MIL-88A	52	18

The concern that large pore sizes may lead to enzyme leakage, while intuitive, but is not supported by our experimental findings or by the broader literature on mesoporous MOF carriers. To address this, we conducted a dedicated enzyme leakage experiment. The immobilized CalB@Meso-MIL-88A was incubated in the reaction buffer under typical reaction conditions (55 °C, 250 rpm) for 24 h. After centrifugation, negligible protein content and enzymatic activity were detected in the supernatant. This confirms that the large pore size does not lead to significant enzyme leakage and that the lipase is stably confined within the W-Meso-MIL-88A framework, likely due to strong multipoint interactions between the enzyme and the carrier surface. In fact, the expansion of pore size is a common strategy to reduce mass transfer limitations and enhance enzymatic activity, and it does not inevitably compromise stability. For instance, Hu et al. reported that lipase was successfully immobilized within macroporous ZIF-8 with pore sizes up to 200 nm, far larger than the enzyme itself. Despite the large pore size, no significant enzyme leakage was observed, and the immobilized lipase maintained over 96% activity after 5 cycles. This was attributed to hydrophobic interactions and the conformational stability provided by the modified pore environment (Hu et al. 2020). Besides, Chen et al. synthesized ultrastable hierarchically porous MOFs (HP-MOFs) using a nucleotide ligand. Their HP-MOFs, featuring micro-, meso-, and macropores used for enzyme immobilization, showed significantly enhanced enzymatic activity and stability, with immobilized lipase retaining over 82% of its initial activity after 6 reuse cycles (Chen et al. 2025). Our results align perfectly with this principle: the combination of a large pore diameter (18 nm) for high accessibility and a favorable chemical environment for strong adsorption in W-Meso-MIL-88A achieves both high activity and exceptional stability against leakage.

Furthermore, the high immobilization efficiency (80–90%) observed not only for CalB but also for other lipases (e.g., Eversa Transform 2.0 (ET 2.0) and *Rhizomucor miehei* Lipase (RML), Fig. S4) suggests the broad potential of W-Meso-MIL-88A as a versatile platform for enzyme immobilization. While the current study focuses on its excellent performance in synthesizing structured phospholipids, this finding opens avenues for its application in other biocatalytic processes.

### CalB@Meso-MIL-88A-catalyzed phosphatidyl EPA/DHA in solvent-free system

**Fig. 3:**
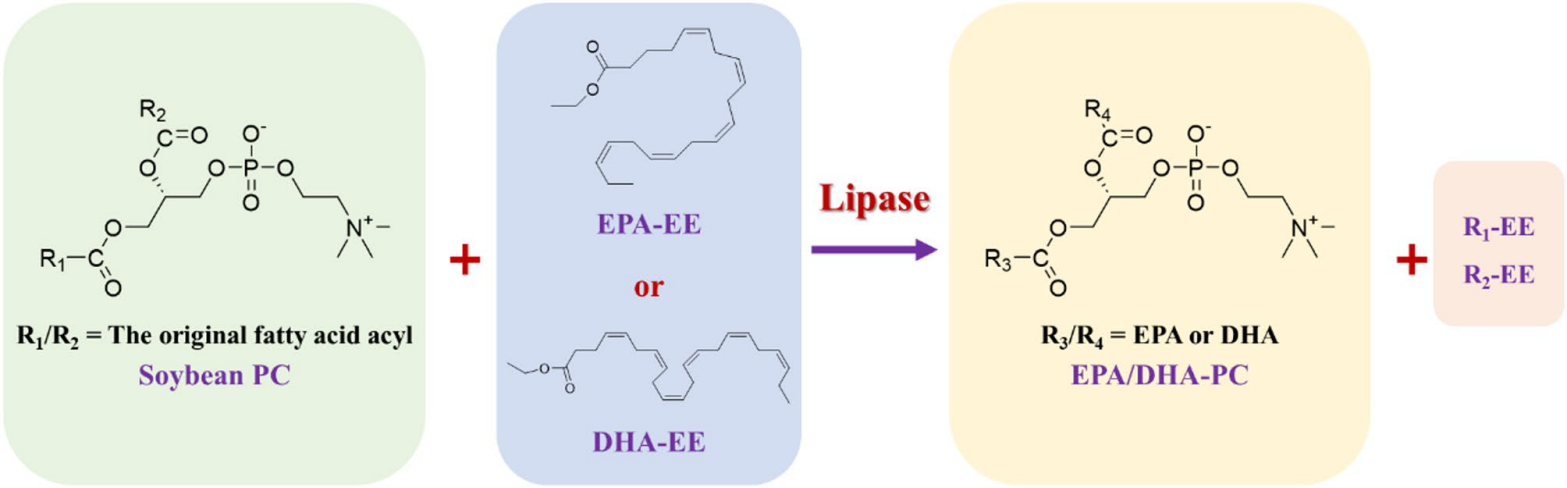
Lipase-catalyzed transesterification of soybean PC with EPA/DHA-EE to synthesize EPA/DHA enriched-PC (phosphatidyl EPA/DHA)

Enzymatic acidolysis of PC with EPA/DHA and transesterification with EPA/DHA-EE have been widely employed to synthesize EPA/DHA-rich PC (phosphatidyl EPA/DHA) (Zhang et al. 2024; Wang et al. 2019; Marsaoui et al. 2015). However, free EPA/DHA are easy to oxidize during the reactions and derived from their EE form, causing a complex preparation process and difficulty preserving for a long time (Vikbjerg et al. 2005; Xi et al. 2016; Li et al. 2016). In addition, EPA/DHA-EE are the main EPA/DHA products in the market (Wang et al. 2019). Hence, enzyme transesterification of PC with EPA/DHA-EE was chosen to synthesize phosphatidyl EPA/DHA, and the relevant reaction scheme is shown in Fig. 3.

**Fig. 4:**
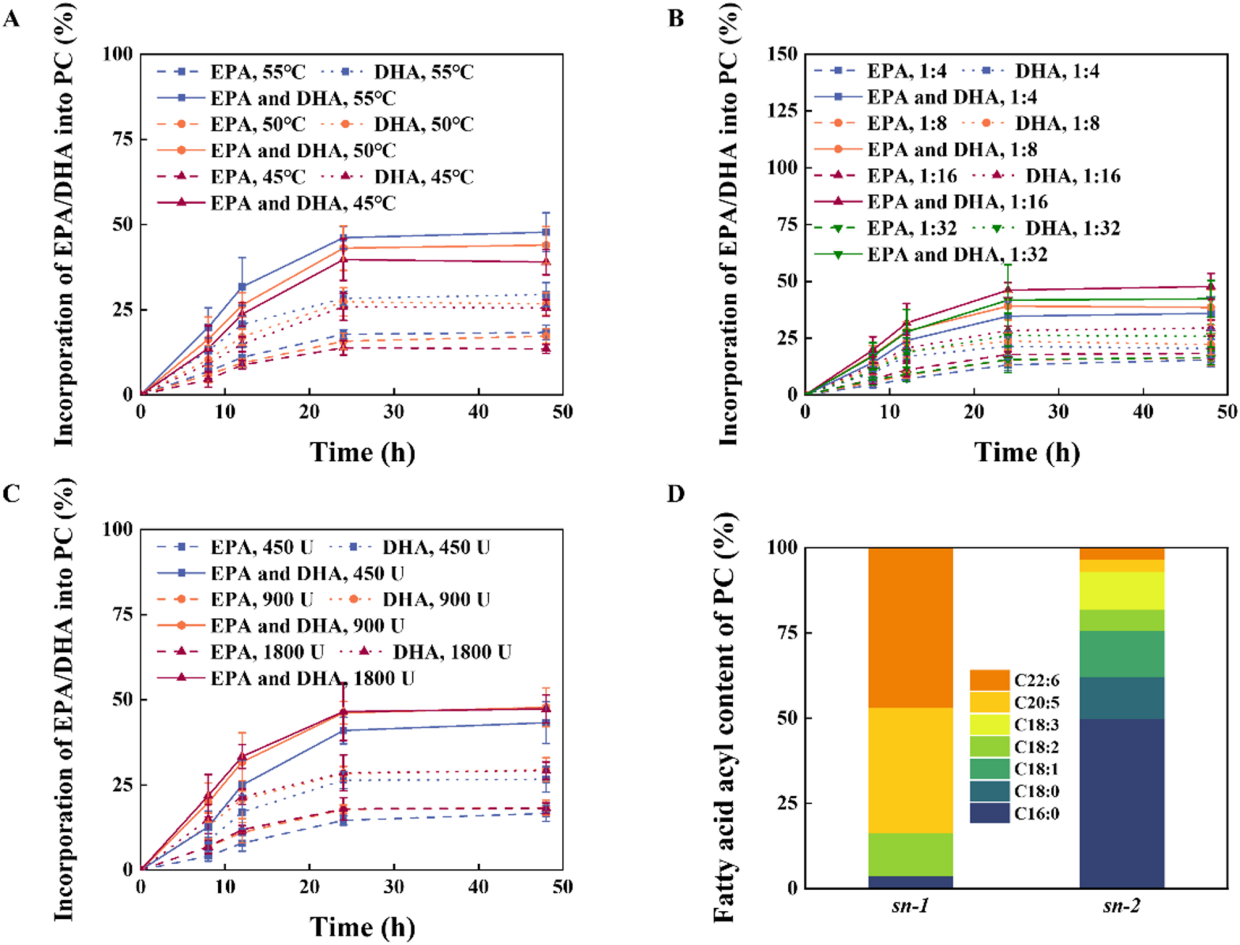
Optimizations of CalB@Meso-MIL-88A-catalyzed phosphatidyl EPA/DHA using 90% EPA/DHA-EE as acyl donor. The effect of **A** the reaction temperature, **B** the molar ratio of EPA/DHA-EE to PC, and **C** the enzyme amount on the transesterification of soybean PC with 90% EPA/DHA-EE. **D** The composition of the fatty acid acyl at the *sn*-1 and *sn-*2 position of phosphatidyl EPA/DHA under the optimal reaction conditions

**Fig. 5:**
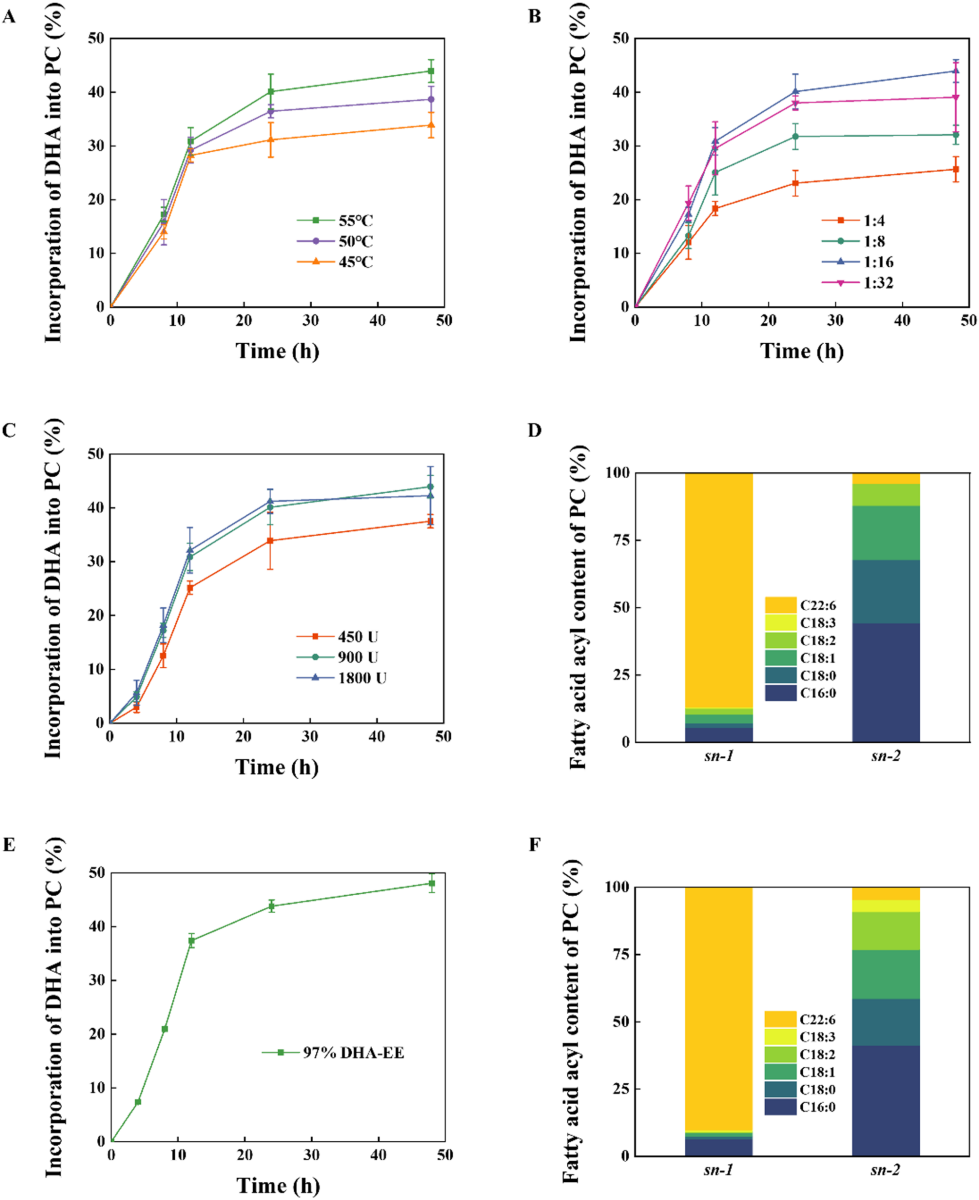
Optimizations of CalB@Meso-MIL-88A-catalyzed phosphatidyl EPA/DHA using 80% DHA-EE or 97% DHA-EE as acyl donor. The effect of **A** the reaction temperature, **B** the molar ratio of DHA-EE to PC, and **C** the enzyme amount on the transesterification of soybean PC with 80% DHA-EE. **D** The composition of the fatty acid acyl at the *sn*-1 and *sn-*2 position of phosphatidyl DHA under the optimal conditions for the reaction of PC with 80% DHA-EE. **E** The total incorporation of DHA into PC and **F** the composition of the fatty acid acyl at the *sn*-1 and *sn-*2 position of phosphatidyl DHA in the reaction of soybean PC with 97% DHA-EE under the optimal conditions mentioned above

Among many lipases and phospholipases, CalB was considered an effective lipase to catalyze the incorporation of EPA, DHA, or some other n-3 polyunsaturated fatty acids (n-3 PUFAs) into phospholipids (Zhang et al. 2024; Chojnacka et al. 2017; Shu et al. 2023). Besides, CalB@Meso-MIL-88A was proved to have a good catalytic effect and reuse performance in the synthesis of phosphatidyl DHA in our previous work (Li et al. 2024). Thus, we carried out a series of optimizations for the transesterification reaction of soybean PC with EPA/DHA-EE catalyzed by CalB@Meso-MIL-88A to achieve a higher total incorporation of EPA/DHA into PC. To simplify the production process, solvent-free system was first chosen. Except for the reaction temperature, the molar ratio of EPA/DHA-EE to PC, and the enzyme amount, which were widely considered as key effects on the reaction (Zhao et al. 2014; Chen et al. 2017), the different initial content of acyl donors, including 90% EPA/DHA-EE (containing 50% EPA-EE and 40% DHA-EE, respectively), 80% DHA-EE and 97% DHA-EE were utilized to achieve a higher yield of phosphatidyl EPA/DHA (Figs. 4 and 5).

While higher temperatures can favor the thermodynamics of endothermic reactions and increase the reaction rate according to Arrhenius’ law, the deactivation of the enzyme at temperatures exceeding its optimum temperature is the primary limiting factor for further improvement (Zhao et al. 2014). The effect of reaction temperature on the total incorporation of EPA/DHA into PC was investigated in the range from 45 to 55 °C, because at less than 40 °C, the substrate mixture could not be stirred sufficiently due to the high viscosity of the mixture, and CalB@Meso-MIL-88A would lose most of its activity when temperature exceeded 60 °C. For these trials, the molar ratio of EPA/DHA-EE or DHA-EE to PC and the enzyme amount were maintained at 16:1 and 900 U (around 5% of total substrate weight), respectively. The results are shown in Figs. 4A and 5A. The total incorporation of EPA/DHA into PC increased with the temperature rising from 45 to 55 °C. Coincidentally, Chojnacka et al. (Chojnacka et al. 2017) and Zhao et al. (Zhao et al. 2014) have reported maximal total incorporation at 55 °C for the transesterification with PC of n-PUFAs, validating the optimal temperature determined in the present study. Thus, 55 °C was selected as the optimal reaction temperature and used in the subsequent experimental trials.

Then, the effects of the EPA/DHA-EE molar ratio to PC on the total incorporation were evaluated from 4:1 to 32:1. Figures 4B and 5B show the results. When the molar ratio varied from 4:1 to 16:1, the total incorporation of EPA/DHA into PC increased. After that, the total incorporation of EPA/DHA into PC decreased when the molar ratio increased from 16:1 to 32:1. The enzyme amount was maintained at 900 U, and the temperature was kept at 55 °C. The maximum total incorporation of EPA/DHA into PC was obtained at the mola ratio of 16:1. This phenomenon can be attributed to the increase of the solubility of PC and the concentration of substrate (EPA/DHA-EE) with the rise of the molar ratio of EPA/DHA-EE to PC from 4:1 to 16:1. When the molar ration was larger than 16:1, the excess of EPA/DHA-EE might decrease mass transfer and inhibit the hydrolytic activity of CalB@Meso-MIL-88A (Shu et al. 2023). Besides, a high substrate molar ratio could lead to difficulties in the separation of the products and increase the cost of the process. Similar results have been reported by Wang et al. (Wang et al. 2019) and Shu et al. (Shu et al. 2023). Hence, EPA/DHA-EE molar ratio was maintained at 16:1 in the subsequent trials.

Enzyme amount ranging from 450 to 1800 U ((around 2.5% to 10% of total substrate weight) was investigated for the transesterification of PC with EPA/DHA-EE. These trails’ temperature and molar ratio were kept at 55 °C and 16:1, respectively. The results are shown in Figs. 4C and 5C. The total incorporation of EPA/DHA into PC increased significantly when the enzyme amount increased from 450 to 900 U. When the enzyme amount reached 1800 U, the total incorporation of EPA/DHA-EE into PC increased slightly. Considering reducing the enzyme amount for economic feasibility, an enzyme amount of 900 U was selected for further trials.

In summary, the optimal temperature, substrate molar ratio, and enzyme amount of the transesterification of PC with EPA/DHA-EE using CalB@Meso-MIL-88A in solvent-free system were 55 °C, 16:1, and 900 U, respectively. Under those conditions, the total incorporation of EPA/DHA into PC for a 48 h reaction with different acyl donors are shown in Table 2. Shu et al. (Shu et al. 2023) and Baeza et al. (Baeza-Jiménez et al. 2012) reported that a substrate with a higher n-PUFA content was conducive to the total incorporation. From the increased total incorporation of DHA into PC with the increase of the content of DHA-EE from 80 to 97%, we could draw a similar conclusion. Positional analysis revealed that the majority of the incorporated EPA/DHA was located at the *sn*-1 position of PC, indicating the specificity of CalB@Meso-MIL-88A towards the *sn*-1 position of PC. Chojnacka et al. (Chojnacka et al. 2017) found a similar phenomenon of the specificity of Novozym 435 lipase (CalB immobilized on macroporous acrylic resin) towards the *sn*-1 position of PC. Compared with the work of Wang et al. (43.55% PUFA-PC) (Wang et al. 2019), Marsaoui et al. (29.1% EPA/DHA-PC) (Marsaoui et al. 2015) and Zhang et al. (39.1% DHA-PC) (Zhang et al. 2023b), where the transesterification took place in solvent-free systems, the total incorporation of EPA/DHA into PC in this work achieved the highest values (48.1% for 97% DHA-EE as acyl donor), indicating the great potential of CalB@Meso-MIL-88A used in the preparation of phosphatidyl EPA/DHA.

**Table 2:** The total incorporation of EPA/DHA into PC and its positional distribution using CalB@Meso-MIL-88A under optimal conditions in solvent-free system

Acyl donor	Total incorporation of EPA/DHA into PC	Distribution of incorporated EPA/DHA at the *sn*-1 position	Distribution of incorporated EPA/DHA at the *sn*-2 position
90% EPA/DHA-EE (50% EPA-EE and 40% DHA-EE)	47.8% (18.3% EPA and 29.5% DHA)	83.6% (36.8% EPA and 46.8% DHA)	6.9% (3.6% EPA and 3.3% DHA)
80% DHA-EE	43.9%	86.9%	3.9%
97% DHA-EE	48.1%	90.1%	4.6%

**Fig. 6:**
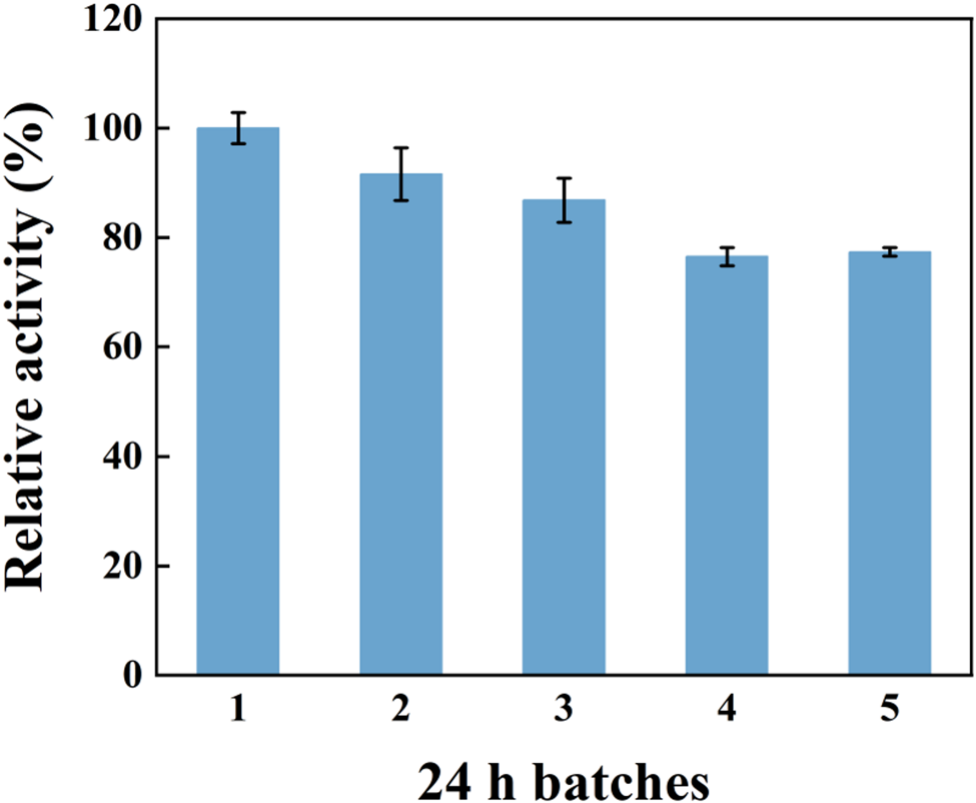
Operational stability of CalB@Meso-MIL-88A. The immobilized lipase was recovered after each batch cycle and reused under identical reaction conditions (55 °C, molar ratio 16:1)

The operational stability, a critical factor for industrial applications, was evaluated under the optimal solvent-free conditions (55 °C, molar ratio 16:1) using 97% DHA-EE as the acyl donor. The catalyst, CalB@Meso-MIL-88A, was recovered by centrifugation after each batch and then introduced into a fresh reaction mixture. As shown in Fig. 6, the immobilized lipase retained 77.3% of its initial activity after 5 consecutive reaction cycles, demonstrating a promising level of robustness for reuse in batch processes. While this stability is promising, further efforts to enhance the recovery efficiency and long-term durability, such as developing magnetic derivatives for easier separation, are underway and will be reported in due course.

### CalB@Meso-MIL-88A-catalyzed phosphatidyl EPA/DHA under different solvent systems

Despite the high total incorporation of EPA/DHA into PC under solvent-free system, the relatively high molar ratio of substrate and long reaction time could be further improved by adding organic solvents. Utilizing organic solvents is necessary to enhance mass transfer and substrate solubility (Marsaoui et al. 2015). Hexane, tert-butanol, and butanone were selected as the solvents for preparing phosphatidyl EPA/DHA using CalB@Meso-MIL-88A. Hexane is the most commonly solvent used in the lipase-catalyzed system (Marsaoui et al. 2015, 2013; Chojnacka et al. 2017; Shu et al. 2023; Estiasih et al. 2020), while tert-butanol (log P = 0.35) and butanone (log P = 0.29) are polar solvents which benefit the solubility of phosphatidylcholine due to its amphipath (Garcia-Quinto et al. 2023). In addition to the type of solvents, the water content caused by different solvents could also affect the total incorporation (Marsaoui et al. 2015; Zhao et al. 2014). To investigate the effect of the water content, 1 g 4 A molecular sieve (MS) activated by drying under vacuum for 2 h at 150 °C was added into different solvent systems (I: hexane, II: hexane: tert-butanol = 1:1, III: hexane: butanone = 1:1). Besides, the molar ratio of EPA/DHA-EE to PC decreased from 16:1 to 9:1, while the reaction temperature and enzyme amount were maintained at 55 °C and 900 U.

**Fig. 7:**
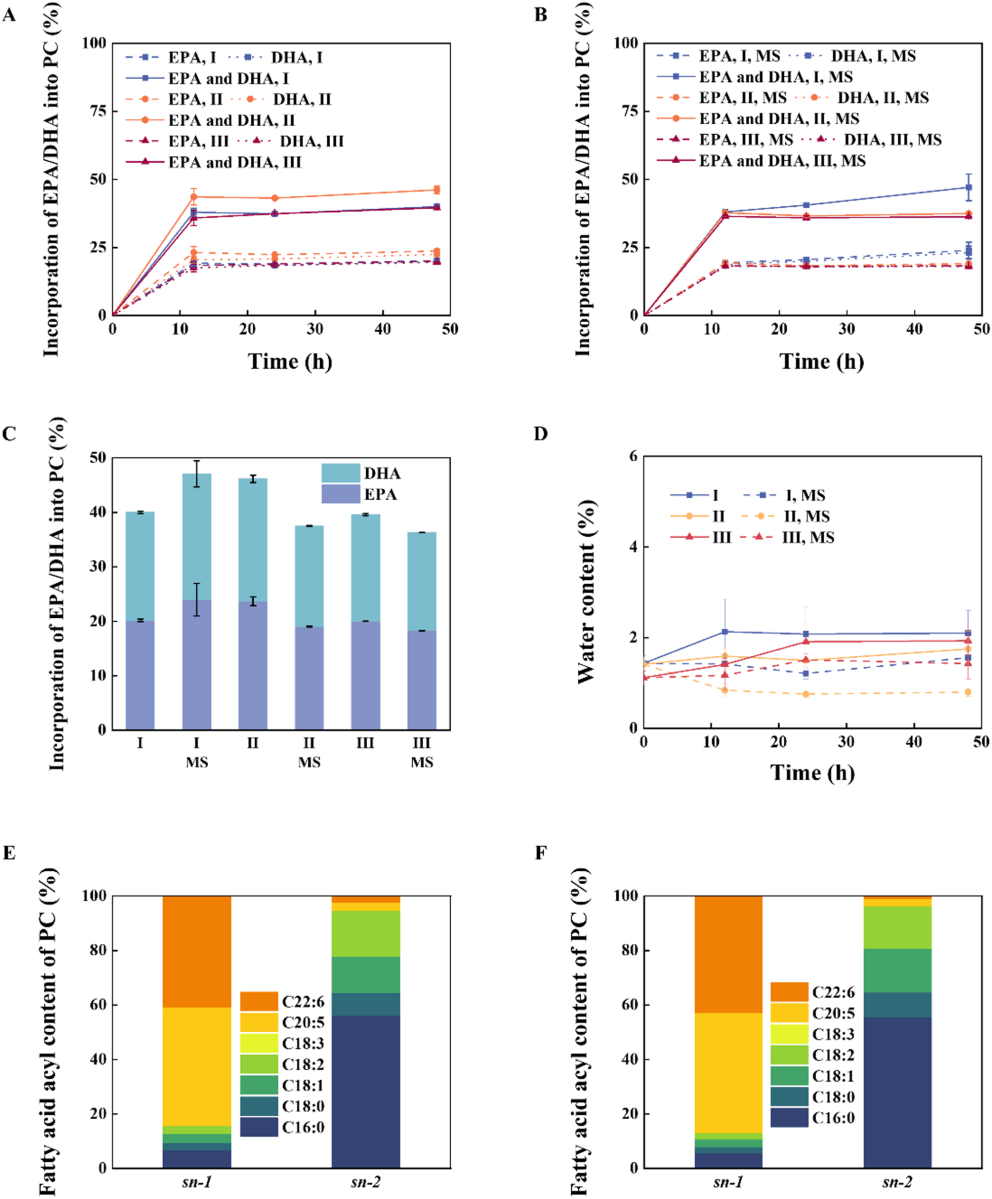
Effects of different organic solvents and the addition of molecular sieve on the total incorporation of EPA/DHA into PC. 90% EPA/DHA-EE was used as an acyl donor. The total incorporation of EPA/DHA into PC **A** without and **B** with 1 g 4A molecular sieve added, where I, II, and III stand for hexane, hexane: tert-butanol = 1:1, and hexane: butanone = 1:1, respectively. **C** The comparison of different reaction systems for 48 h, and MS stands for molecular sieve added. **D** The water content of different systems during the reaction. The composition of the fatty acid acyl at the *sn*-1 and *sn-*2 position of phosphatidyl EPA/DHA under **E** hexane with MS system and **F** hexane: tert-butanol = 1:1 without MS system

The comparisons of the total incorporation of EPA/DHA into PC under different solvent systems are shown in Figs. 7A–C and S5A–C. The two highest total incorporations were achieved in system I (hexane) with MS and system II (hexane: tert-butanol = 1:1) without MS. The total incorporation of system III (hexane: butanone = 1:1) with MS or not was not satisfactory. Through experimental observation, the solubility of PC into system III was harder than that of the other two systems, resulting in the low total incorporation of EPA/DHA into PC. As shown in Figs. 7D and S5D, the initial water content of system I (hexane) was higher than that of system II (hexane: tert-butanol = 1:1). After adding 1 g 4A MS, the water content of system I was closer to that of system II without MS, indicating the optimal water content was around 1.5% for 90% EPA/DHA-EE (Fig. 7D) and 1.9% for 80% DHA-EE (Fig. S5D). Besides, most of EPA/DHA was incorporated into the *sn*-1 position of PC (Figs. 7E–F and S5E-F), consistent with the results of solvent-free system. The transesterification of PC with 97% DHA-EE was conducted under system I with MS and system II without MS as shown in Fig. S7. The total incorporation of EPA/DHA into PC under different solvent systems are summarized in Table S1. Introducing an organic solvent effectively reduced the substrate molar ratio, enhanced the reaction rate, and maintained high levels of total incorporation.

Table S2 summarizes the results of synthesizing phosphatidyl EPA/DHA using the transesterification strategy of PC with n-3 PUFA esters in solvent systems. The total incorporation of EPA/DHA into PC in this work also achieved high levels, indicating the practical applicability of CalB@Meso-MIL-88A in preparing phosphatidyl EPA/DHA under solvent-free and solvent systems.

### Kinetic study and molecular docking simulation of enzyme

As shown in Tables 2 and S1, different total incorporations and reaction rates of EPA/DHA into PC were obtained under different reaction systems. Besides, the preference of CalB@Meso-MIL-88A to EPA or DHA changed with the transform from solvent-free system (18.3% EPA and 29.5% DHA total incorporation into PC) to solvent systems (24.0% EPA and 23.1% DHA total incorporation into PC in hexane, 23.7% EPA and 22.5% DHA total incorporation into PC in hexane and tert-butanol mixture). To investigate the mechanism, the kinetic study of CalB@Meso-MIL-88A for the transesterification of PC with EPA-EE and DHA-EE under different systems and the molecular docking simulation between CalB and EPA/DHA-EE were conducted.

**Fig. 8:**
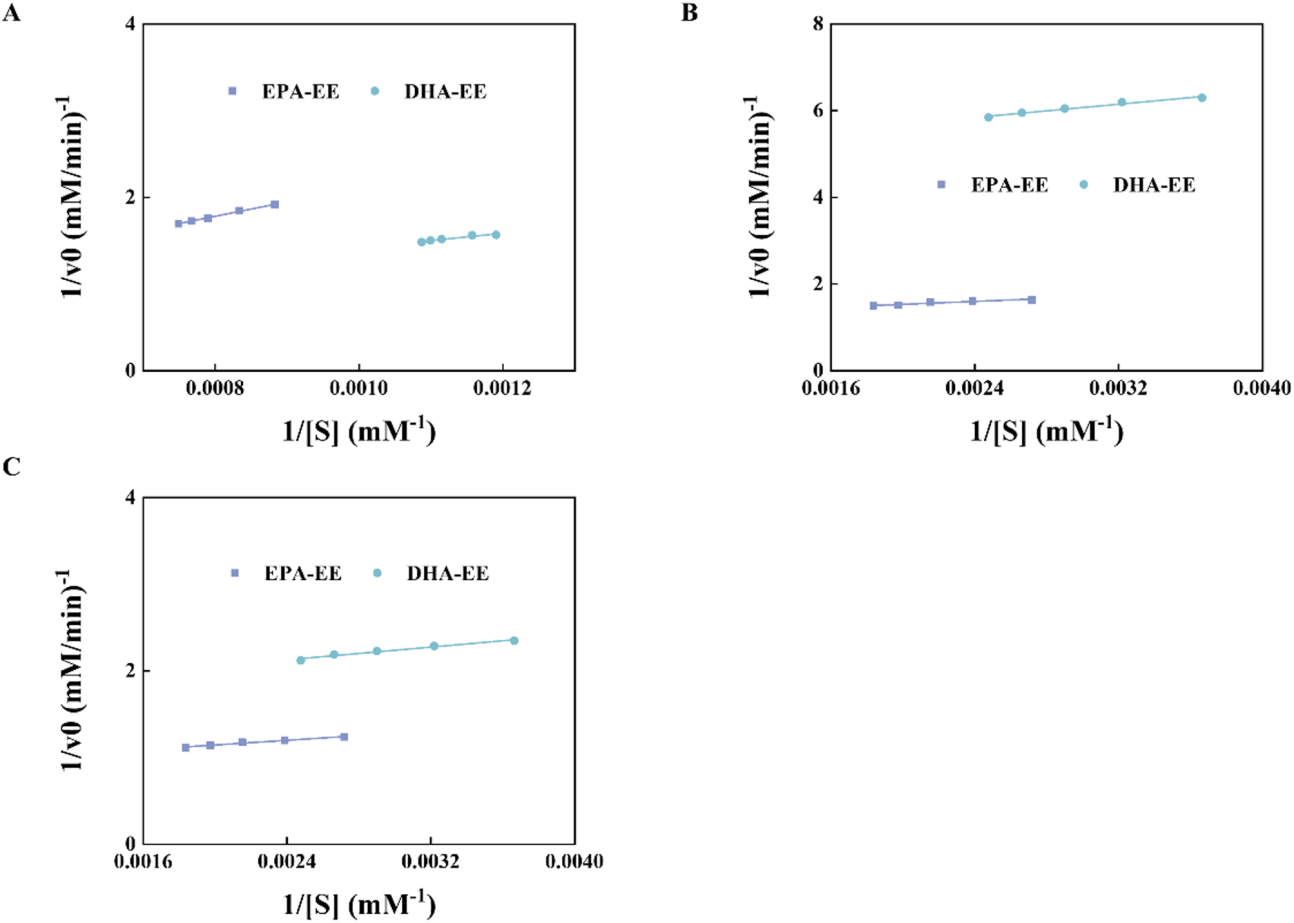
Lineweaver–Burk plots of CalB@Meso-MIL-88A for the transesterification of PC with EPA-EE and DHA-EE in **A** solvent-free system, **B** system I (hexane with MS) and **C** system II (hexane: tert-butanol = 1:1, without MS)

Michaelis–Menten parameters provide a good insight into the kinetic behavior of enzymes (Prabhavathi Devi et al. 2009). To eliminate the mass transfer effect of external diffusion and ensure that CalB@Meso-MIL-88A was saturated by the substrate, the rotational speed and the enzyme amount were first optimized, as shown in Fig. S7. Under any system, the rotational speed at 250 rpm and the enzyme amount at 360 U were suitable. Besides, the reaction conversion for the first 30 min was used to calculate the initial reaction rate because it was in the straight-line region. Table 3 shows *K*_m_, *V*_max_, *K*_cat_, and *K*_cat_/ *K*_m_ of CalB@Meso-MIL-88A in the transesterification of PC with EPA-EE and DHA-EE, calculated by Lineweaver–Burk plots as shown in Fig. 8. The Michaelis–Menten constant (*K*_m_) represents the enzyme’s affinity for its substrate, where lower values indicate higher affinity. The turnover number (*K*_cat_) showcases the ability of the enzyme to convert substrate into product per time. The *K*_cat_/ *K*_m_ ratio has been used as a measure index of enzyme performance (Jang et al. 2023).

**Table 3:** Michaelis–Menten parameters of CalB@Meso-MIL-88A for the transesterification of PC with EPA-EE and DHA-EE under different systems

System	Substrate	*V*_max_ mM/min	*K*_m_ mM	*K*_cat_ min^−1^	*k*_cat_/*K*_m_ mM^−1^ min^−1^
Solvent-free	EPA-EE	2.242	3741.93	5.99E-04	1.60E-07
	DHA-EE	1.689	1396.10	1.21E-03	8.66E-07
System I with MS	EPA-EE	0.836	140.43	5.96E-03	4.24E-05
	DHA-EE	0.203	77.56	2.62E-03	3.37E-05
System II without MS	EPA-EE	1.145	154.94	7.39E-03	4.77E-05
	DHA-EE	0.593	108.67	5.45E-03	5.02E-05

Compared with solvent-free system, *K*_cat_/ *K*_m_ in system I and system II were increased by two orders of magnitude, indicating that CalB@Meso-MIL-88A had a more significant and efficient conversion efficiency in the presence of solvents. In any system, the *K*_m_ value of DHA-EE was lower than that of EPA-EE, indicating that CalB@Meso-MIL-88A had a stronger affinity for DHA-EE. The difference between the two was more evident in solvent-free system, resulting in a relatively higher *K*_cat_ of DHA-EE. This might be the main reason why, in solvent-free system, the incorporation of DHA was higher than that of EPA, although the initial concentration of EPA-EE was a little higher than that of DHA-EE. Marsaoui et al. (Marsaoui et al. 2013) have reported a similar phenomenon, where 11.32% EPA and 12.30% DHA were incorporated into PC with 52% EPA-EE and 20% DHA-EE added.

**Fig. 9:**
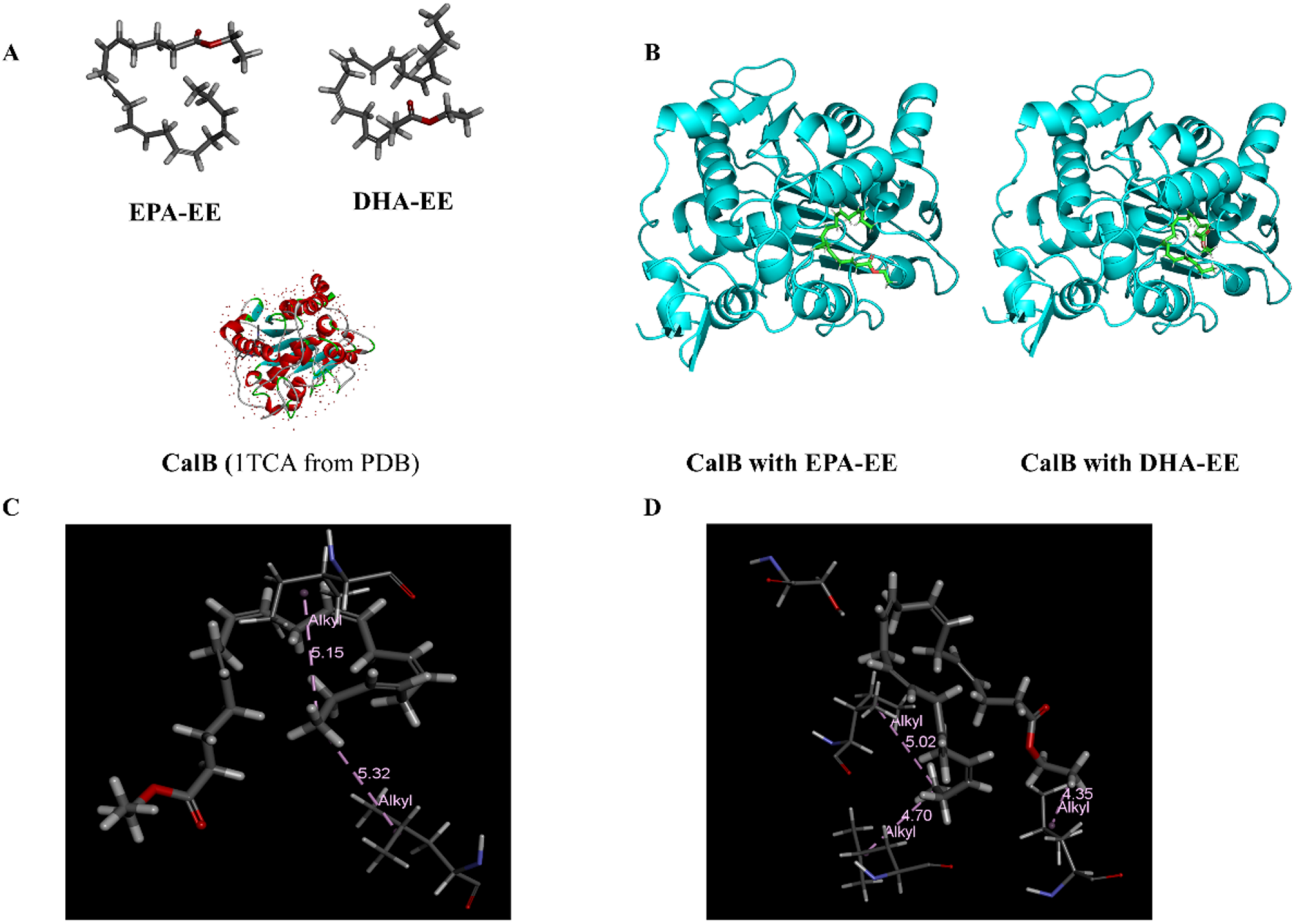
Molecular docking simulation of CalB with EPA-EE and DHA-EE. **A** The structures of the ligands (EPA-EE and DHA-EE) and protein receptor (CalB, 1TCA from PDB). **B** The visualized results of molecular docking using Flexible Docking. **C** The interactions between CalB and EPA-EE. **D** The interactions between CalB and DHA-EE

The molecular docking simulation was conducted to investigate why CalB has a stronger substrate affinity and conversion efficiency for DHA-EE than that of EPA-EE. Firstly, the CHARMm force field was applied to ligands (EPA-EE and DHA-EE), and internal energy was released through Minimize Ligands. The CalB crystal structure was taken from the Protein Data Bank (PDB entry: 1TCA). The natural ligand molecules and all water molecules were removed first to reduce the amount of simulation calculation. Then, hydrogen atoms were supplemented, and the protein structure was checked. To prepare the receptor 1TCA, the active site region was determined through the calculation, and the radius was set to 12 A to ensure that EPA/DHA-EE molecules could be fully covered in this region. Flexible Docking was chosen, where the conformation of protein side chains and ligands can be freely changed during the docking process, which is mainly used to examine the binding mode between molecules accurately. However, in practical applications, only the residual side chain at the active site is generally defined as flexible due to the computational amount and calculation time. In Flexible Docking, ligands will be attached to receptors as follows: select the receptor flexible amino acid residues and calculate a set of protein conformations by changing the side chain conformation using CHARMm; use LibDock to attach flexible ligand pairs to the active site of each receptor conformation; use CHARMm to optimize selected protein side chains in the presence of rigid ligands; optimize the final ligand docking conformation using CDOCKER. In the output result, CDOCKER Interaction Energy refers to the non-bonding interaction between proteins and ligands, which is generally negative (Ning et al. 2021). The greater the absolute value, the better the binding between proteins and ligands and the higher the binding energy, which is conducive to reducing the activation energy of the reaction and improving the reaction rate (Oger et al. 2010). We selected the docking scenario with the highest LidDockScore for EPA-EE (91.6681) and DHA-EE (91.7714) docking with CalB as the premise for comparison. The simulation results showed that the CDOCKER Interaction Energy value of DHA-EE (-45.0998 kcal/mol) was higher than that of EPA-EE (− 38.3718 kcal/mol) in absolute value. CalB had better selectivity for DHA-EE, consistent with the previous experiment, which showed that *K*_m_ was smaller for DHA-EE in different systems. The visualized results of CalB docking with EPA-EE and DHA-EE are shown in Fig. 9B–D show that the main interactions between CalB and the two ethyl ester substrates are hydrophobic interaction and intermolecular van der Waals force interaction.

Taken together, the kinetic studies and molecular docking simulations provide consistent and complementary evidence for the higher affinity of CalB towards DHA-EE over EPA-EE. The significantly lower Michaelis constant (*K*_m_) for DHA-EE across all reaction systems (Table 3) indicates a stronger enzyme–substrate binding affinity. This experimental finding is well corroborated by the molecular docking results, which showed a more favorable (more negative) CDOCKER Interaction Energy for DHA-EE (− 45.10 kcal/mol) compared to EPA-EE (− 38.37 kcal/mol), suggesting a more stable binding conformation within the catalytic pocket of CalB. This synergistic effect of a lower energy barrier (docking) and higher binding affinity (kinetics) ultimately contributes to the observed preference for DHA incorporation.

## Conclusion

Meso-MIL-88A was synthesized via a soft-template method with citrate dissociation, using ethanol/water eluents. Nitrogen adsorption–desorption and XPS analyses confirmed tunable pore sizes and sulfur residue levels dependent on eluent composition. The immobilized CalB@Meso-MIL-88A exhibited high catalytic efficiency for EPA/DHA incorporation into phosphatidylcholine, achieving 86.8% *sn*-1 positional specificity (44.2% EPA, 42.7% DHA) with 90% EPA/DHA-ethyl ester donors, and 90.1% of the incorporated DHA residing at the sn-1 position with 97% DHA-ethyl ester—among the highest reported performances to date. Kinetic and molecular docking studies revealed stronger substrate affinity for DHA-ethyl ester, driven by enhanced transfer efficiency in organic solvents compared to solvent-free systems. This work highlights the industrial viability of Meso-MIL-88A for lipase immobilization and positions CalB@Meso-MIL-88A as a robust biocatalyst for structured phospholipid synthesis.

## Supplementary Information

Below is the link to the electronic supplementary material.

Supplementary Material 1

## Data Availability

All data generated during this study are included in this article.
