# Supplementary material for: Enzymatic synthesis of phosphatidyl-EPA/DHA using Candida antarctica lipase B immobilized on mesoporous MIL-88 A
Source: Bioresour Bioprocess. 2025 Nov 3;12(1):123. doi: 10.1186/s40643-025-00959-5 (PMC12583253; doi:10.1186/s40643-025-00959-5)
Supplement: Supplementary file 1 — Supplementary Material 1 [file 40643_2025_959_MOESM1_ESM.docx]

**SUPPORTING INFORMATION**

**Enzymatic Synthesis of Phosphatidyl-EPA/DHA using *Candida antarctica* Lipase B Immobilized on Mesoporous MIL-88A**

Yuhan Li, Guowei Wu, Zeqing Liu, Lingmei Dai, Dehua Liu, Wei Du*

Key Laboratory for Industrial Biocatalysis, Ministry of Education,

Department of Chemical Engineering, Tsinghua University, Beijing 100084, China

^*^Corresponding author: [duwei@tsinghua.edu.cn](mailto:duwei@tsinghua.edu.cn)

**SUPPLEMENTARY EXPERIMENTAL SECTION**

**Synthesis of E-Meso-MIL-88A**

348 mg (3 mmol) of fumaric acid (FA) and 2.50 g sodium dodecyl sulfate (SDS) were dissolved in 140 mL deionized water and preheated at 55 °C with agitation at 250 rpm for 1 h to form a stable micellar system. Subsequently, 270 mg (1 mmol) of FeCl_3_·6H_2_O was dissolved in 10 mL of deionized water and added to the above mixed solution. The reaction proceeded at 55 °C and 250 rpm in a shaker for 24 h. Then, the light reddish-brown precipitates were collected by centrifugation at 8000 rpm for 3 min and immersed into 150 mL of ethanol via ultrasonication in an ultrasonic bath under 300 W and 40 kHz for approximately 6 h to remove SDS and any unreacted precursor solution. After thorough washing and centrifugation, the precipitates were dried under a vacuum at 60 °C overnight. Then, 100 mg of the resultant powder was dispersed in 50 mL of deionized water, and 10 mL of 0.02 mol/L sodium citrate solution was added to the system. The mixture was maintained at 30 °C for 1 h to dissociate the part of the outer MOFs and expose the internal pores. The resulting product after dissociating was denoted as E-Meso-MIL-88A.

**Synthesis of W-Meso-MIL-88A**

The basic synthesis process of W-Meso-MIL-88A was similar to that of E-Meso-MIL-88A, except that the washing process used deionized water instead of ethanol.

**Determination of lipase loading**

The enzyme loading was determined by measuring the difference in protein concentration using the Bicinchoninic Acid Assay (BCA) Protein Assay Kit between the original solution and the remaining protein content in the supernatant after immobilization, as described by Eq (1).

| $L\left( Enzyme Loading \right)=\frac{(C_{0}-C_{1})\times V}{m_{s}}$ | (1) |
| --- | --- |

Where $C_{0}$ and $C_{1}$ are the protein concentrations of the supernatant before and after the immobilization (mg/mL), respectively. $V$ is the volume of total solution (1 mL), and $m_{s}$is the weight of carrier (50 mg around).

All data presented are average results of triplicated repetition of each run.

**Determination of lipase activity and acticity recovery**

Free and immobilized lipase activity was obtained using the tributyrin hydrolysis method. For free lipase, 1 mL of 98% tributyrin and 3 mL of 0.05 M phosphate buffer (PB buffer) (pH = 7.0) were preheated and pre-emulsified at 40 ℃ and 200 rpm for 15 minutes. Subsequently, 10 μL of thirty times diluted (30x) CalB solution was added to the reaction medium. After incubating for 10 minutes, 10 mL of 95% ethanol was added to the reaction solution to deactivate the enzyme. The amount of tributyl acid produced was titrated with a standard solution of NaOH (0.05 M). For immobilized lipase, 1 mg around of CalB@Meso-MIL-88A was utilized, and the general procedure remained the same as for free lipase, except that the quantities of reactants were scaled up to 12 mL of 98% tributyrin and 24 mL of buffer. Immobilized lipase was filtrated through a 0.2 μm filter membrane to terminate the reaction. Eq (2) and Eq (3) below were employed to calculate the specific activity of free and immobilized lipase, respectively.

| $U_{F}=\frac{(V_{1}-V_{0})\times C_{\mathrm{NaOH}}}{m_{F}\times t}$ | (2) |
| --- | --- |
| $U_{IL}=\frac{(V_{1}-V_{0})\times C_{\mathrm{NaOH}}}{m_{IL}\times\frac{L}{L+1}\times t}$ | (3) |

Where *U* is the specific activity (μmol∙min^-1^∙mg^-1^ or U/mg), with $U_{F}$ and $U_{IL}$ representing the specific activity of the free and immobilized lipase, respectively, $V_{1}$ is the volume of NaOH titrated (mL) when lipase (free or immobilized) is used, $V_{0}$ is the titrated volume (mL) when the blank is used, $C_{\mathrm{NaOH}}$ is the concentration of sodium hydroxide (0.05 M), $m_{F}$ and $m_{IL}$ are the masses of free or immobilized lipase (mg) respectively, $L$ is the enzyme loading (mg/g) of immobilized lipase and $t$ is the reaction time (10 min).

The activity recovery was then calculated using Eq (4).

| $R \left( \% \right)=\frac{U_{IL}}{U_{F}}\times100$ | (4) |
| --- | --- |

All data presented are average results of triplicated repetition of each run.

**Analysis of fatty acid composition by gas chromatograph (GC)**

A 30 μL sample was withdrawn from the reaction mixture at different times for analysis. The samples were washed with acetone twice to extract the ethyl ester mixture, and the acetone was removed from the sample by spin evaporation under 60 ℃ for 30 minutes. Then, a 10 μL sample was added to a solution of 600 μL heptadecanoic acid methyl ester in ethanol solution (internal standard, 100 mg/100 mL), and 0.5 μL of the resulting mixture was injected into an Agilent 7890 gas chromatograph (GC) equipped with CP-FFAP capillary column (0.32 mm×0.30 μm×25 m). The initial column temperature was set at 180 °C and maintained for 0.5 min, followed by heating to 250 °C at a rate of 10 °C/min and held for 6 min. The detector and injector were set at 260 °C and 250 °C, respectively. The conversion of ethyl ester was calculated using Eq (5):

| $Y \left( conversion of ethyl ester,\% \right)=\frac{(\frac{m_{i}}{m_{0}}\times\frac{A_{0}}{A_{i}}-\frac{m_{i}}{m_{t}}\times\frac{A_{t}}{A_{i}})}{\frac{m_{i}}{m_{t}}\times\frac{A_{t}}{A_{i}}}\times100$ | (5) |
| --- | --- |

Where $m$ stands for the mass, and $A$ stands for the GC peak area. The subscripts *i*, *0* and *t* stand for internal standard, ethyl ester before and after reaction, respectively. All data presented are average results of triplicated repetition of each run. The total incorporation of EPA/DHA into PC is $\frac{x}{2}$ times the corresponding conversion of ethyl ester. For instance, if the conversion of DHA-EE was 10% when the addition molar ratio of DHA-EE to PC was 8:1, the total incorporation of DHA into PC would be 40%.

**Analysis of fatty acid composition in the *sn*-1 and *sn*-2 position of PC**

The analysis of fatty acid composition in the *sn*-1 and *sn*-2 position of PC referred to the work of Kielbowicz et al.^1^. At the end of the reaction, around 50 mg product PC was dissolved by 2 mL 95% ethanol and 1 mL chloroform. Then 0.4 g Lipozyme TL IM was added to the mixture to hydrolyze or ethanol hydrolyze *sn*-1 fatty acid acyl of PC and shaken vigorously under 45 ℃ and 250 rpm for 8 h. After the enzyme was filtered off, ethanol was removed at 45 ℃ on a rotary evaporator in vacuo. Subsequently, 3 mL of a chloroform-methanol solution (2:1, *v/v*) was added, and the organic layer was separated. The products were then analyzed by thin layer chromatography (TLC) (chloroform: methanol: water, 65:25:4 *v/v/v*) and evaporated at 60 °C in vacuo. The bands of free fatty acids and ethyl esters were at the top of the plate, representing the fatty acids at the *sn*-1 position of the product PC, while the band of *sn*-2 lysophosphatidylcholine (*sn*-2 LPC) was at the middle of the plate representing the fatty acids at the *sn*-2 position of the product PC. The bands mentioned above were collected, and the methylation of the samples was conducted using the BF_3_-MeOH method, as outlined in studies by He et al. ^2^ and Kim et al.^3^. The resultant fatty acid methyl esters were then measured by GC with the procedure as mentioned above to calculate the content of each fatty acid acyl at the *sn*-1 and *sn*-2 position of the product PC.


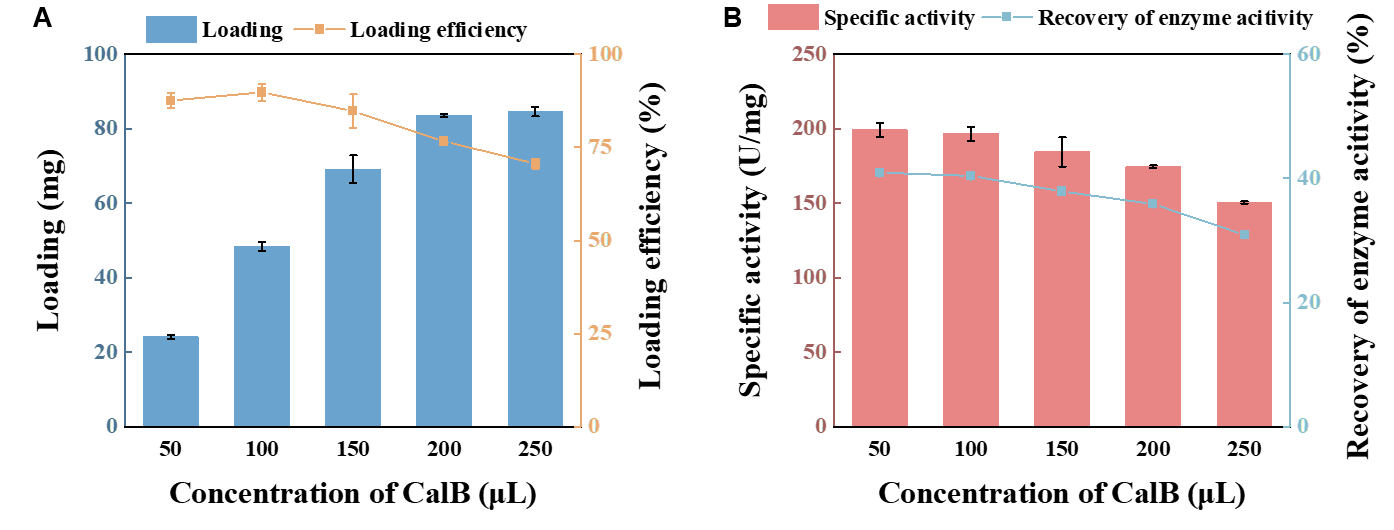


**Fig. S1.** Optimization of CalB concentration for immobilization on Meso-MIL-88A. (A) Loading and loading efficiency, (B) specific activity and recovery of enzyme activity of immobilized CalB on Meso-MIL-88A with different concentrations of CalB added. The concentration of 200 μL was selected for all subsequent experiments as it provided the best balance of high activity recovery and immobilization efficiency.


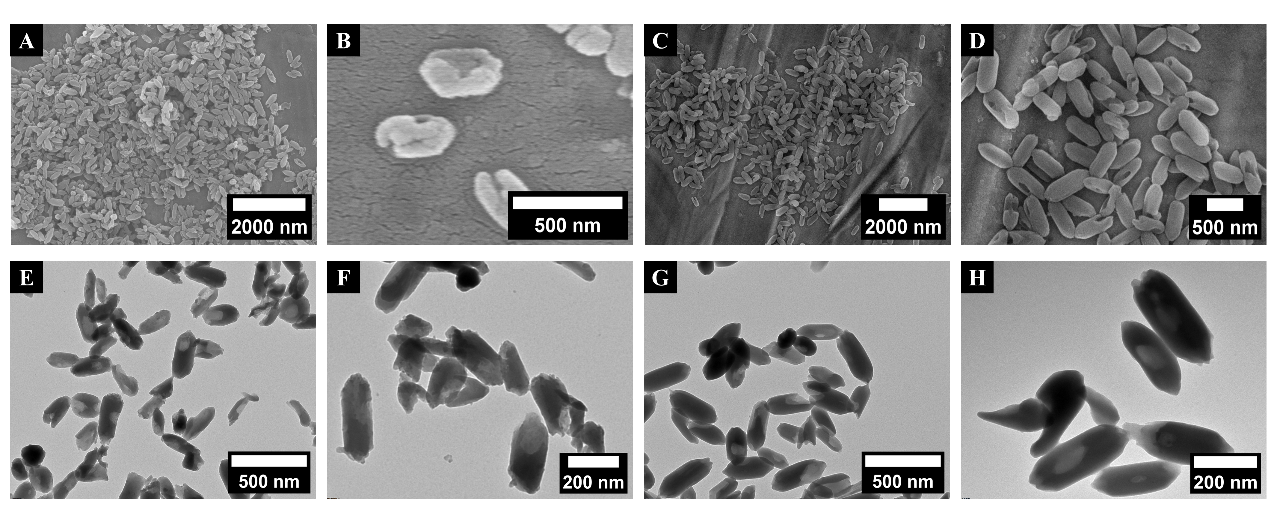
**Fig. S2.**  SEM and TEM images of W-Meso-MIL-88 A and E-Meso-MIL-88A. (A) and (B) are the SEM images of W-Meso-MIL-88A. (C) and (D) are the SEM images of E-Meso-MIL-88A. (E) and (F) are the TEM images of W-Meso-MIL-88A. (G) and (H) are the TEM images of E-Meso-MIL-88A.

**
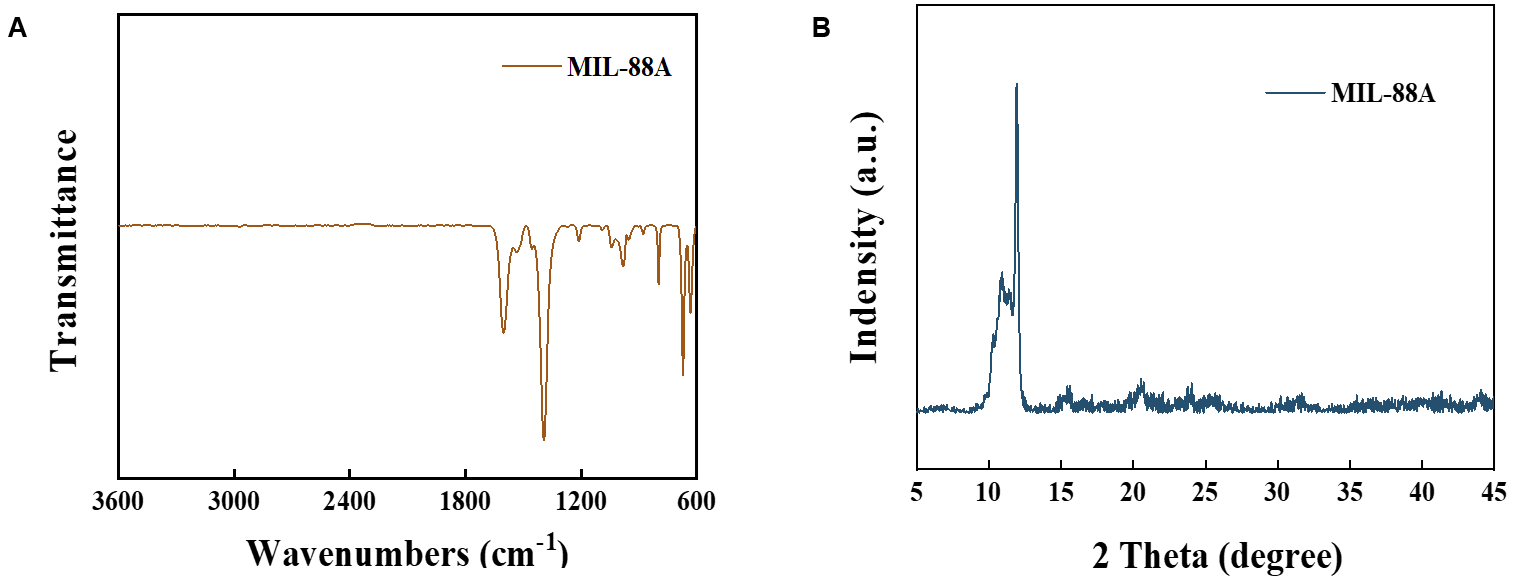
**

**Fig. S3.**  (A) ATR-FTIR pattern and (B) XRD pattern of MIL-88A.

**
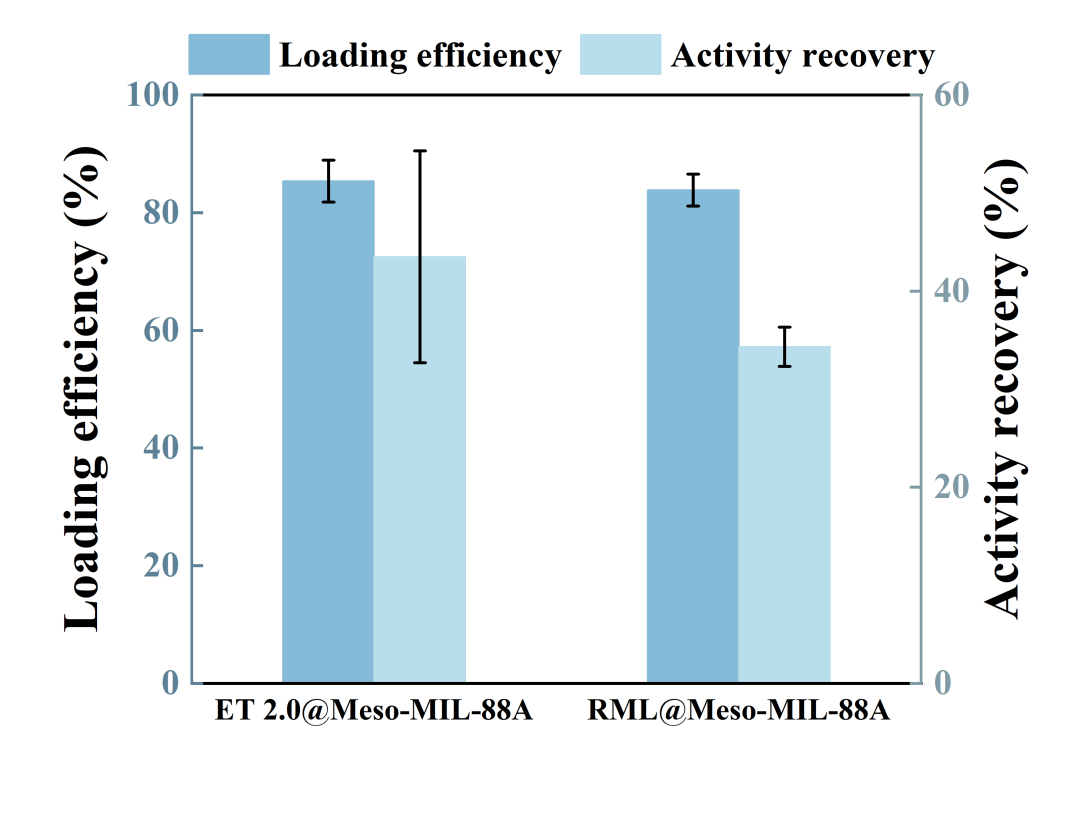
**

**Fig. S4.** Loading efficiency and activity recovery of immobilized Eversa Transform 2.0 (ET 2.0) and *Rhizomucor miehei* Lipase (RML) on W-Meso-MIL-88A.


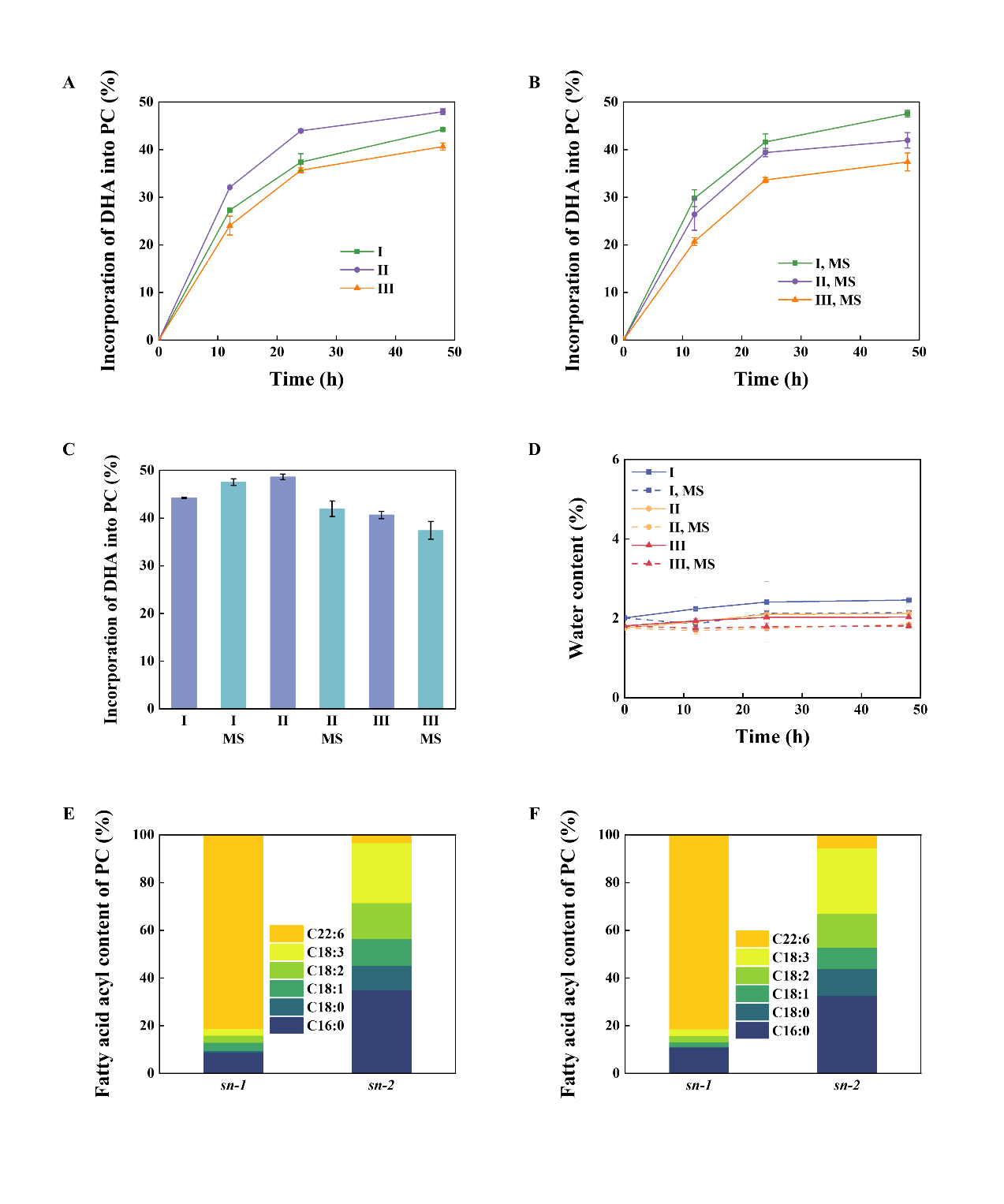


**Fig. S5.** Effects of different organic solvents and the addition of molecular sieve on the total incorporation of DHA into PC. 80% DHA-EE was used as an acyl donor. The total incorporation of DHA into PC (A) without and (B) with 1 g 4A molecular sieve (MS) was added, where I, II, and III stand for hexane, hexane: tert-butanol=1:1, and hexane: butanone=1:1, respectively. (C) The comparison of different reaction systems for 48 hours, and MS stands for molecular sieve added. (D) The water content of different systems during the reaction. The composition of the fatty acid acyl at the *sn*-1 and *sn-*2 position of phosphatidyl DHA under (E) the hexane with MS system and (F) the hexane: tert-butanol=1:1 without MS system.


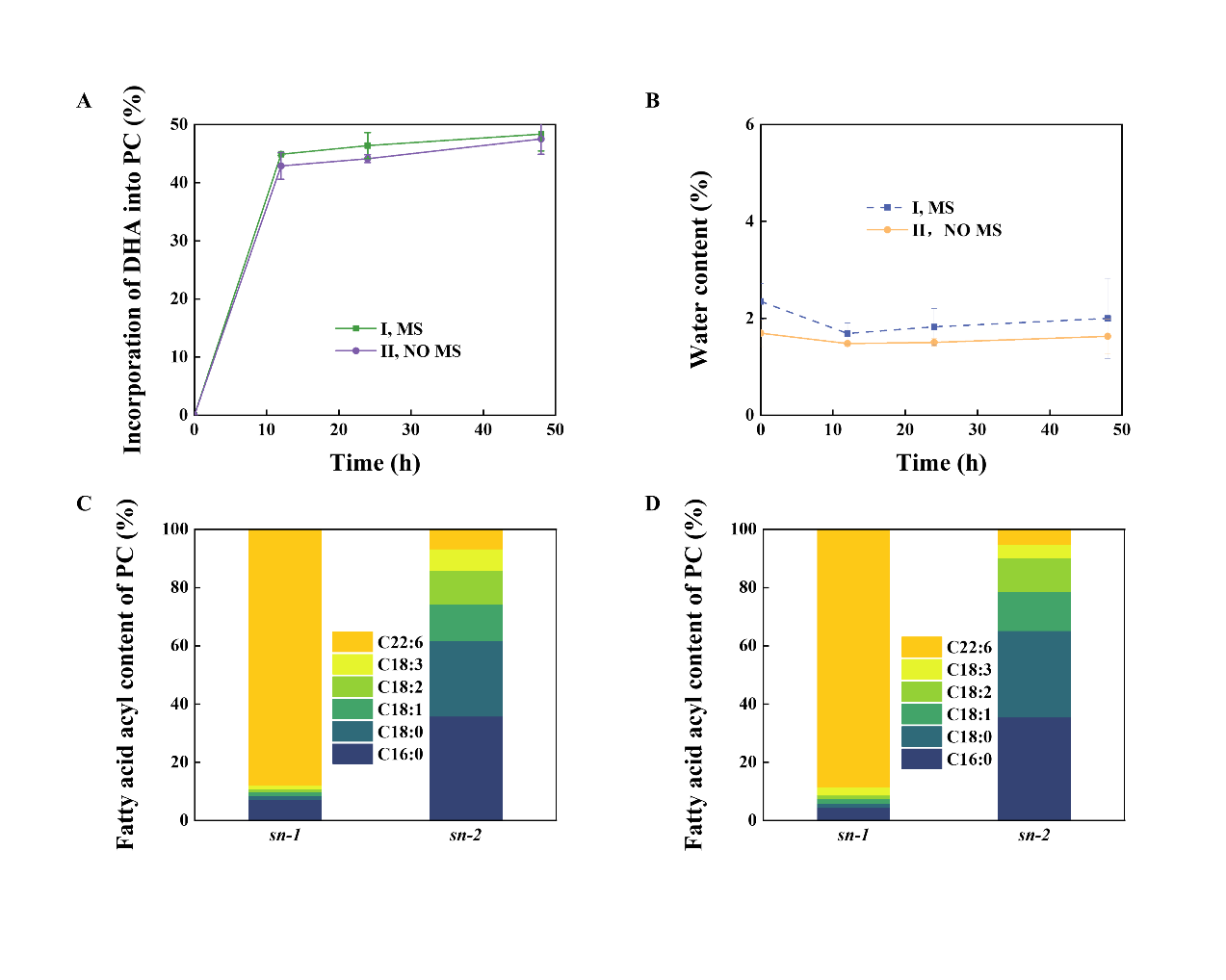


**Fig. S**6**.** Effects of different organic solvents and the addition of molecular sieve on the total incorporation of DHA into PC. 97% DHA-EE was used as an acyl donor. (A) The total incorporation of DHA into PC under the hexane with MS system (I, MS) and the hexane: tert-butanol=1:1 without MS system (II, NO MS). (B) The water content of those two systems during the reaction. The composition of the fatty acid acyl at the *sn*-1 and *sn-*2 position of phosphatidyl DHA under (C) the hexane with MS system and (D) the hexane: tert-butanol=1:1 without MS system.


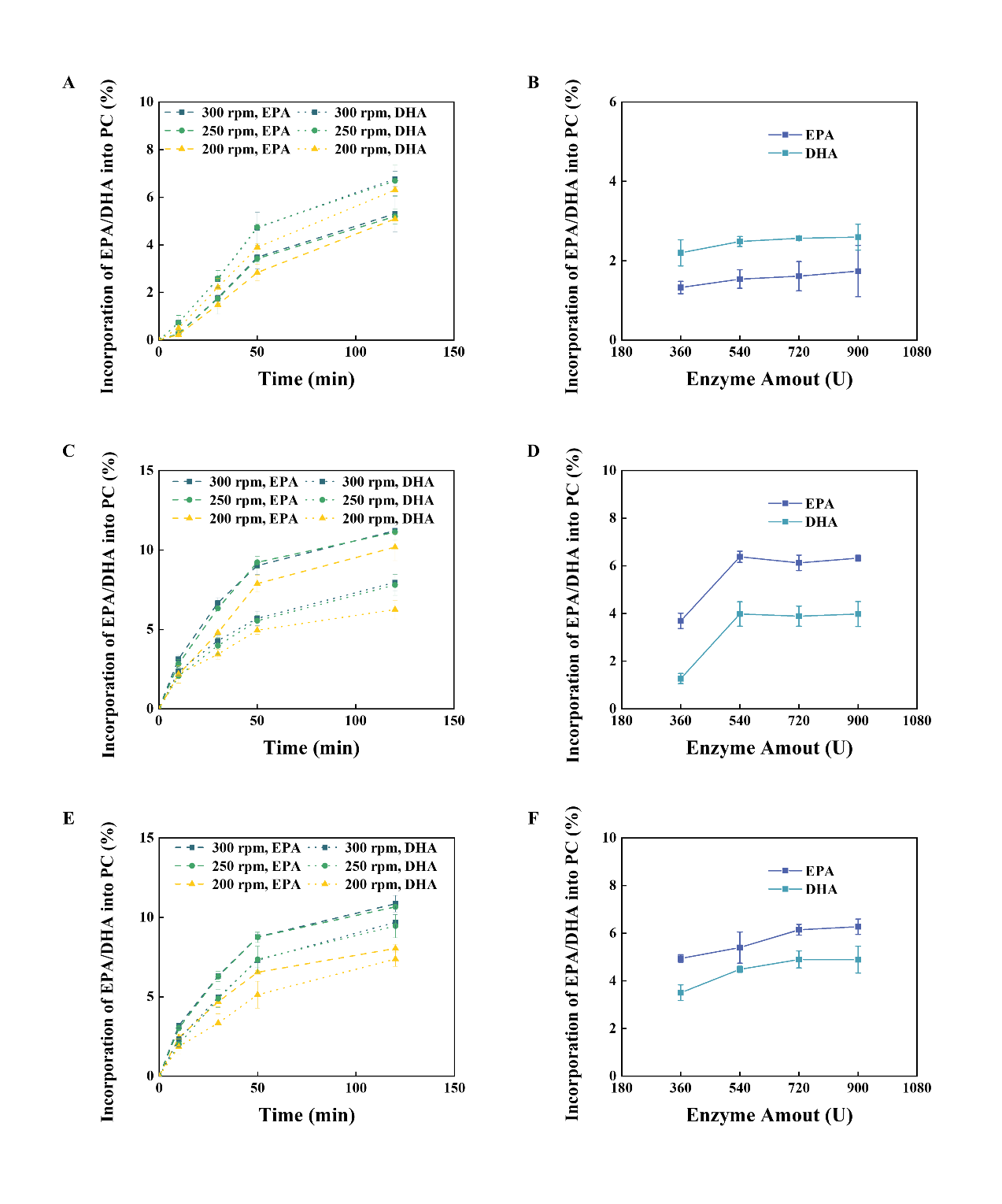


**Fig. S7.** The screening of the rotational speed and the enzyme amount for the kinetic study. (A) and (B) for a solvent-free system, (C) and (D) for system I (hexane with MS), and (E) and (F) for system II (hexane: tert-butanol=1:1, without MS).

**Table S1.** The total incorporation of EPA/DHA into PC and its positional distribution using CalB@Meso-MIL-88A under the optimal conditions under solvent systems.

| **Acyl donor** | **Solvent system** | **Total incorporation of EPA/DHA into PC** | **Distribution of incorporated EPA/DHA at the *sn*-1 position** | **Distribution of incorporated EPA/DHA at the *sn*-2 position** |
| --- | --- | --- | --- | --- |
| 90% EPA/DHA-EE  (50% EPA-EE and 40% DHA-EE) | hexane with 1 g 4A MS | 47.1%  (24.0% EPA and 23.1% DHA) | 84.4%  (43.6% EPA and 40.7% DHA) | 5.3%  (3.0% EPA and 2.3% DHA) |
| 80% DHA-EE | hexane with 1 g 4A MS | 47.5% | 81.3% | 3.3% |
| 97% DHA-EE | hexane with 1 g 4A MS | 48.4% | 87.9% | 6.7% |
| 90% EPA/DHA-EE  (50% EPA-EE and 40% DHA-EE) | hexane: tert-butanol=1:1 | 46.2%  (23.7% EPA and 22.5% DHA) | 86.8%  (44.2% EPA and 42.7% DHA) | 3.6%  (2.5% EPA and 1.0% DHA) |
| 80% DHA-EE | hexane: tert-butanol=1:1 | 47.9% | 81.4% | 5.4% |
| 97% DHA-EE | hexane: tert-butanol=1:1 | 47.5% | 88.5% | 5.2% |

**Table S2**. The synthesis of phosphatidyl EPA/DHA using the transesterification strategy of PC with esters in solvent systems.

| **Enzyme** | **Acyl donor** | **Solvents** | **Temperature** | **Substrate ratio** | **Enzyme amount** | **Time** | **Yield** | **Reference** |
| --- | --- | --- | --- | --- | --- | --- | --- | --- |
| Immobilized CalB | n-3 PUFA-EE  (48% DHA) | hexane | 50 ℃ | 8:1 (mass) | 25% | 72 h | 31.2% DHA-PC | ^4^ |
| *Rhizomucor miehei* lipase | 52% EPA-EE and 20% DHA-EE | hexane with Mg^2+^/urea | 50 ℃ | 3:1 (mass) | 30% | 72 h | 56.8%  EPA/DHA-PC | ^5^ |
| *Rhizomucor miehei* lipase | 52% EPA-EE and 20% DHA-EE | hexane with Mg^2+^/Ca^2+^ | 50 ℃ | 2:1 (mass) | 10% | 24 h | 11.32% EPA-PC  12.30% DHA-PC | ^6^ |
| Lipozyme TL IM | 10% EPA-EE and 70% DHA-EE | heptane 8  isooctane 1  hexane 1 | 55 ℃ | 2:1 (mass) | 25% | 5.5 h | 22.99% DHA/EPA-PC | ^7^ |
| Lipase OF from *C. rugosa* | 10.1% EPA-EE and 68.5% DHA-EE | hexane | 37 ℃ | 7:1 (molar) | 2.2×10^5^ U  / 2 mmol Soy PL | 72 h | 47.1% PUFA-PC | ^8^ |
| Novozym 435 | 26.7% EPA-EE and 45.2% DHA-EE | hexane | 55 ℃ | 10:1 (molar) | 20% | 48 h | 45.6% PUFA-PC  (36.8% DHA-PC  5.8% EPA-PC) | ^9^ |

**References**

(1) Kielbowicz, G.; Gladkowski, W.; Chojnacka, A.; Wawrzenczyk, C. A Simple Method for Positional Analysis of Phosphatidylcholine. *Food Chem.* **2012**, *135* (4), 2542-2548. DOI: 10.1016/j.foodchem.2012.07.005.

(2) He, C.; Cao, J.; Bao, Y.; Sun, Z.; Liu, Z.; Li, C. Characterization of Lipid Profiling in Three Parts (Muscle, Head and Viscera) of Tilapia (Oreochromis Niloticus) Using Lipidomics with UPLC-ESI-Q-TOF-MS. *Food Chem.* **2021**, *347*, 129057. DOI: 10.1016/j.foodchem.2021.129057.

(3) Kim, I. H.; Garcia, H. S.; Hill, C. G. Synthesis of Structured Phosphatidylcholine Containing n‐3 PUFA Residues via Acidolysis Mediated by Immobilized Phospholipase A_1_. *J. Am. Oil Chem. Soc.* **2010**, *87* (11), 1293-1299. DOI: 10.1007/s11746-010-1609-7.

(4) Shu, L. W.; Zheng, X.; Qi, S. H.; Lin, S. Z.; Lu, Y. H.; Yao, C. Y.; Ling, X. P. Transesterification of Phosphatidylcholine with DHA-Rich Algal Oil Using Immobilized *Candida antarctica* Lipase B to Produce DHA-Phosphatidylcholine. *Enzyme Microb. Technol.* **2023**, *169*, 110266. DOI: 10.1016/j.enzmictec.2023.110266.

(5) Marsaoui, N.; Naghmouchi, K.; Baah, J.; Raies, A.; Laplante, S. Incorportation of Ethyl Esters of EPA and DHA in Soybean Lecithin Using *Rhizomucor miehei* Lipase: Effect of Additives and Solvent-Free Conditions. *Appl. Biochem. Biotechnol.* **2015**, *176* (3), 938-946. DOI: 10.1007/s12010-015-1621-3.

(6) Marsaoui, N.; Laplante, S.; Raies, A.; Naghmouchi, K. Incorporation of Omega-3 Polyunsaturated Fatty Acids into Soybean Lecithin: Effect of Amines and Divalent Cations on Transesterification by Lipases. *World J. Microbiol. Biotechnol.* **2013**, *29* (12), 2233-2238. DOI: 10.1007/s11274-013-1388-z.

(7) Zhan, Z. Assistant of Ultrasonic Irradiation Synthesize Phospholipids Enriched with n-3 Polyunsaturated Fatty Acids by Lipase. *J. Chin. Cereals Oils Assoc.* **2011**, *26* (11), 42-46. DOI: 1003-0174(2011)26:11<42:CSFZZF>2.0.TX;2-6.

(8) Yamamoto, Y.; Mizuta, E.; Ito, M.; Harata, M.; Hiramoto, S.; Hara, S. Lipase-Catalyzed Preparation of Phospholipids Containing n-3 Polyunsaturated Fatty Acids from Soy Phospholipids. *J. Oleo Sci.* **2014**, *63* (12), 1275-1281. DOI: 10.5650/jos.ess14125.

(9) Chojnacka, A.; Gładkowski, W.; Grudniewska, A. Lipase-Catalyzed Transesterification of Egg-Yolk Phophatidylcholine with Concentrate of n-3 Polyunsaturated Fatty Acids from Cod Liver Oil. *Mol.* **2017**, *22* (10), 1771. DOI: 10.3390/molecules22101771.
